# Bacterial Community Structural Dynamics and Predicted Functional Potential in *Loxostege sticticalis*

**DOI:** 10.3390/insects17101068

**Published:** 2026-10-15

**Authors:** Cheng Ye, Yuxuan Chen, Huimin Gu, Yunxia Cheng, Dianjie Xie, Zhong Liang, Jun Lin, Xuan Li, Lei Zhang, Xingfu Jiang, Hailong Kong

**Affiliations:** 1 College of Plant Protection, Yangzhou University, Yangzhou 225009, China; akuyca@yeah.net (C.Y.); 15050735334@163.com (Y.C.); 19505271767@163.com (H.G.); 2 State Key Laboratory for Biology of Plant Diseases and Insect Pests, Institute of Plant Protection, Chinese Academy of Agricultural Sciences, No. 2 of West Yuanmingyuan Road, Beijing 100193, China; yxcheng@ippcaas.cn (Y.C.); xiedianjie@caas.cn (D.X.); 3 Center for Grassland Biological Disaster Prevention and Control of Xinjiang Uygur Autonomous Region, No. 69 Heilongjiang Road, Urumqi 830000, China; 18999280970@163.com (Z.L.); xjcy2009@163.com (J.L.); xnlixuan@126.com (X.L.)

**Keywords:** *Loxostege sticticalis*, symbiotic bacteria, microbial community diversity, dynamic change, potential function

## Abstract

Symbiotic bacteria contribute to insect physiology and environmental adaptation, yet their dynamics and potential functions in the major migratory pest *Loxostege sticticalis* remain poorly understood. We characterized bacterial community composition, diversity, and predicted functional potential across developmental stages, tissues and frass samples, and environmental conditions. In laboratory-reared insects, *Enterococcus* and *Staphylococcus* predominated from the second to fifth instars, whose predicted functions emphasized carbohydrate and nucleotide metabolism. Adults were instead dominated by *Pseudoalteromonas* and *Asaia*, with predicted profiles showing greater representation of lipid and amino acid metabolism and transport and catabolism. First-instar larvae exhibited an adult-like community structure. Among sample types, *Enterococcus* predominated in the midgut, whereas *Staphylococcus* was overrepresented in the epidermis and frass. Epidermal and frass communities emphasized cellular processes and genetic information processing, whereas midgut and other internal tissue communities showed greater predicted potential for membrane transport and metabolism. Wild-caught larvae harbored distinct communities consistently enriched in *Stenotrophomonas* and *Ralstonia*, with predicted profiles shifting toward carbohydrate, amino acid, and lipid metabolism and environmental adaptation in later instars. Overall, these findings demonstrate that the symbiotic bacterial communities of *L. sticticalis* vary markedly with development, tissue niche, and environment, providing a basis for further studies of host–microbe interactions.

## Simple Summary

The beet webworm, *Loxostege sticticalis*, is a major migratory crop pest, but little is known about its bacterial dynamics and functions during development or in different environments. We examined bacterial communities across developmental stages, tissues and frass samples, and laboratory and field conditions. In laboratory-reared insects, *Enterococcus* and *Staphylococcus* were most abundant from the second through fifth larval stages, whereas *Pseudoalteromonas* and *Asaia* became dominant in adults. Predicted functions in these larval stages were mainly related to carbohydrate use and genetic information processing, whereas adults showed greater potential for fat and amino acid metabolism and substance transport. Newly hatched larvae had bacterial communities similar to those of adults. *Enterococcus* predominated in the midgut, while *Staphylococcus* was more abundant in body-surface tissues and frass. Functional predictions suggested greater potential for bacterial growth and processing of genetic information in body-surface tissues and frass, but for nutrient metabolism and transport in the gut and other internal tissues. Wild larvae contained distinct communities dominated by *Stenotrophomonas* and *Ralstonia* with predicted functions shifting toward nutrient metabolism in later stages. These findings show that bacterial communities in *L. sticticalis* change with development, tissues and frass, and environmental conditions, providing insights into potential microbial roles in host adaptation.

## 1. Introduction

Microorganisms inhabit nearly all organisms and constitute their microbiota [1]. In insects, these communities colonize internal and external surfaces and can support host physiology, adaptation, and survival under diverse environmental conditions [2,3]. These microorganisms can also shape host immunity and interactions with pathogens [4]. Insect-associated microbial communities typically account for approximately 1–10% of total host biomass. Insects can therefore be regarded as multiorganismal entities [5]. The insect microbiota includes bacteria, symbiotic fungi, viruses, and other microorganisms. However, bacteria generally dominate in both abundance and functional contribution [6]. These bacteria are involved throughout the insect life cycle and influence a wide range of host traits [6]. Their contributions to nutrient acquisition, microbial competition, and responses to pathogens can affect host performance [3,6]. Because bacterial partners can also alter insecticide susceptibility, these interactions provide potential targets for pest management [7,8].

The insect gut is a major habitat for symbiotic bacteria [9]. Its bacterial community can change across developmental stages in response to multiple factors [10,11]. For example, *Chironomus transvaalensis* harbors a distinct bacterial community at each developmental stage, including the egg, larval, pupal, and adult stages [12]. In *Oulema melanopus*, *Wolbachia* sp. and *Lactococcus lactis* constitute a large proportion of the adult microbiome. These taxa are markedly less abundant in larvae, which instead harbor a more diverse bacterial community. Such stage-specific differences are largely driven by feeding habits, which affect microbial colonization, abundance, and diversity [13]. Similarly, gut bacterial diversity varies across the developmental stages of *Helicoverpa armigera* [14] and other lepidopterans [15]. Stage-associated changes have also been reported in *Telchinia issoria*, with diversity varying during larval development and metamorphosis [16]. Differences in diet and host physiology may contribute to microbial variation among lepidopterans [17,18].

Insect-associated bacteria occur at internal sites beyond the gut, including the hemocoel, salivary glands, and specialized host cells [13,19,20,21]. Bacterial communities at different body sites generally differ in both composition and function. Surface- or exoskeleton-associated bacteria are often involved in defense or communication [22,23]. By contrast, gut communities contribute to nutrient digestion and absorption, essential vitamin and amino acid synthesis, and physiological homeostasis [24]. This functional heterogeneity reflects structural differences among bacterial communities, as distinct tissues within an insect can harbor different assemblages [25]. However, comparative evidence for compositional and functional differences among tissues remains limited in lepidopteran insects [23,26].

The composition and functional potential of the insect gut microbiota are strongly influenced by external factors, including diet, microbial exposure, and habitat heterogeneity [27]. Laboratory-reared insects are typically maintained under controlled, homogeneous conditions with simplified diets, limited microbial inputs, and reduced environmental stressors. Wild populations, by contrast, continuously encounter complex, dynamic environments and diverse environmental microbial reservoirs [28,29]. These contrasting conditions can produce pronounced differences in gut microbial community structure between laboratory-reared and wild insects. Wild cockroach populations, for example, harbor greater gut microbial alpha diversity than laboratory-reared populations. Moreover, their alpha diversity decreases markedly after laboratory rearing [29]. Field crickets similarly exhibit greater microbial diversity and distinct gut community structures in wild populations than in laboratory populations [28]. In Spodoptera litura, different plant and artificial diets were associated with changes in gut bacterial composition, metabolite abundance, and microbiota–metabolome relationships [30].

*Loxostege sticticalis* L. (Lepidoptera: Pyraloidea: Crambidae) is a migratory crop pest distributed across northern China, North Asia, North America, and Eastern Europe [31]. In China, *L. sticticalis* was included in the Ministry of Agriculture and Rural Affairs’ 2023 list of Class I crop pests [32]. Through diapause and long-distance migration, this pest damages a wide range of crops over broad geographical areas and infests 259 host plant species [31,33]. During outbreaks, aggregating larvae consume leaves, stems, and reproductive organs along their migration routes. The resulting crop losses can reach 60% and, in severe cases, cause complete crop failure [34]. Studies of the *L. sticticalis* microbiota have mainly focused on comparisons of gut microbial communities in relation to diapause [35,36]. However, the broader organization and functional roles of symbiotic microbiota remain poorly understood.

To investigate the compositional dynamics and potential functions of symbiotic bacterial communities in *L. sticticalis*, we performed high-throughput 16S rRNA gene sequencing. We characterized the bacterial community composition and functional potential across developmental stages, tissue and frass samples, and environmental conditions. The findings provide a foundation for future investigations of host–symbiont interactions and for evaluating potential pest management strategies that target symbiotic bacteria.

## 2. Materials and Methods

### 2.1. Insect Rearing

Laboratory populations of *Loxostege sticticalis* larvae were provided by the Institute of Plant Protection, Chinese Academy of Agricultural Sciences. Adults received a 10% honey solution, whereas larvae were fed fresh leaves of their natural host plant, lambsquarters (*Chenopodium album* L.). Both life stages were maintained in an environmental chamber at 22 ± 1 °C and 70 ± 5% relative humidity under a 16:8 h light:dark photoperiod.

### 2.2. Collection of Loxostege sticticalis Across Developmental Stages, Tissues, and Field Conditions

Newly emerged males and females were paired to establish five independent female lineages, and offspring from each lineage were reared separately. Larvae from the same lineage that hatched within 24 h were transferred to sterilized 300 mL plastic containers. Larvae were fed fresh *Chenopodium album* leaves, and sterilized containers were used to reduce contamination from the rearing environment. Midgut samples were collected on the first day of each larval instar, from the first through fifth instars, during pupation, and on the third day of the adult stage. Larval instars were identified from head capsule width and body morphology. For each larval instar, the pupal stage, and each adult sex, one pooled sample was prepared from each of the five female lineages, resulting in five biological replicates per group. Each replicate consisted of midguts pooled from 5 to 15 individuals originating from the same female lineage. The number of individuals per replicate varied with body size to ensure sufficient biological material for DNA extraction. Each pooled sample was considered one biological replicate.

For gut sampling, first- to fifth-instar larvae, pupae, and female and male adults were surface-sterilized and dissected under sterile conditions in phosphate-buffered saline (PBS). Larvae were sequentially treated with 75% ethanol and 5% NaClO solution, placed on ice, and decapitated before dissection. Pupae were opened after surface sterilization, and adults were sprayed with ethanol before dissection. The intact midgut and its contents were carefully removed from each individual using sterile forceps. Fifth-instar larvae were also used for the comparison of midgut, epidermis, other internal tissues, and frass samples. “Other internal tissues” comprised the remaining internal tissues after removal of the midgut and epidermis. Epidermis samples consisted of the body covering remaining after internal tissues were removed. This fraction was rinsed with PBS, and residual internal tissues were removed. For each of the three tissue types, four biological replicates were prepared, with the corresponding tissues from five larvae pooled per replicate. Frass was collected 6 h after individual fifth-instar larvae were placed in sterilized plastic containers under regular feeding conditions. Four biological replicates were prepared for frass, each consisting of frass pooled from ten larvae. Samples of different types originated from different insects. All midgut, tissue, and frass samples were immediately stored at −80 °C until DNA extraction.

For the wild population, larvae from the first through fifth instars were collected in the field in May 2023. Each instar comprised five biological replicates, each consisting of midguts pooled from 5 to 15 individuals. The number of individuals per pool varied with body size to obtain sufficient biological material and DNA for sequencing. Wild larvae were collected from *Chenopodium album* plants in Zhengxiangbai Banner, Xilin Gol League, Inner Mongolia Autonomous Region, China (42.210876° N, 114.891301° E). Larvae were collected alive and taken indoors for processing. After dissection, midguts were immediately stored at −80 °C and submitted to Novogene for sequencing.

### 2.3. High-Throughput Sequencing of Bacterial 16S rRNA Genes

Genomic DNA was extracted by Beijing Novogene Bioinformatics Technology Co., Ltd., (Beijing, China) using the cetyltrimethylammonium bromide (CTAB) method. Total DNA extracted from the *L. sticticalis* samples was used as the template for PCR amplification. The V3–V4 region of the bacterial 16S rRNA gene was amplified using the forward primer 5′-CCTAYGGGRBGCASCAG-3′ and reverse primer 5′-GGACTACNNGGGTATCTAAT-3′. Sample-specific barcodes were incorporated into the primers used for amplification. PCR began with denaturation at 98 °C for 1 min. Thirty cycles followed, each comprising 98 °C for 10 s, 50 °C for 30 s, and 72 °C for 30 s. A final extension was performed at 72 °C for 5 min. PCR negative controls containing double-distilled water (ddH_2_O) instead of template DNA were included in all sequencing projects. Experimental samples were processed further only when no amplification band was detected in the negative controls by agarose gel electrophoresis. The PCR negative controls were also sequenced by Beijing Novogene Bioinformatics Technology Co., Ltd.; however, the corresponding sequencing outputs were retained by the company and were not included in the datasets available for downstream analysis. No DNA extraction blanks were included.

PCR products were verified by electrophoresis on 2.0% agarose gels and purified using the Universal DNA Purification and Recovery Kit (Beijing Novogene Co., Ltd., Beijing, China). Purified amplicons were quantified and pooled at equal mass before library preparation. Indexed sequencing libraries for wild-larva samples were prepared using the NEBNext^®^ Ultra™ II FS DNA PCR-free Library Prep Kit (New England Biolabs, Ipswich, MA, USA; Cat. No. E7430L), whereas libraries for laboratory developmental-stage and tissue/frass samples were prepared using the NEBNext^®^ Ultra™ II DNA Library Prep Kit (New England Biolabs, USA; Cat. No. E7645B), following the sequencing provider’s workflow. The libraries were quantified using a Qubit^®^ 2.0 Fluorometer (Thermo Fisher Scientific Inc., Waltham, MA, USA). Libraries that passed quality control were sequenced on the Illumina NovaSeq 6000 platform by Beijing Novogene Bioinformatics Technology Co., Ltd.

### 2.4. Statistical and Bioinformatic Analyses

Reads were demultiplexed by barcode, and barcode and primer sequences were removed. Paired-end reads were merged using FLASH v1.2.11 (minimum overlap, 10 bp; maximum mismatch density, 0.2). Cutadapt v3.3 was used to trim sequences beyond reverse-primer matches and remove sequences shorter than 20 nt. Merged sequences were quality-filtered using fastp v0.23.1 (-q 19; -u 15), and reference-based chimeras were removed using VSEARCH v2.16.0 against SILVA. The retained merged sequences were imported into QIIME 2 v2022.2.1 as single-end sequences and denoised using the DADA2 denoise_single action (q2-dada2 plugin v2022.2.0; trim_left = 0, trunc_len = 0, max_ee = 2, trunc_q = 2). An additional consensus chimera-removal step was performed within DADA2, with min_fold_parent_over_abundance = 1. Each distinct denoised sequence was defined as an amplicon sequence variant (ASV). Taxonomy was assigned against SILVA v138.1 using the QIIME 2 classify-sklearn naive Bayes classifier with a confidence threshold of 0.7. Per-sample sequencing counts and rarefaction depths are provided in Supplementary Table S1. Before subsequent analyses in NovoMagic V3.0 (Novogene, Beijing, China), ASVs assigned to Cyanobacteria were removed using the platform’s taxonomic filtering tool. This filtering step also removed ASVs annotated as unidentified_Chloroplast within Cyanobacteria. The filtering criterion was applied to the ASV table, and relative abundances were expressed as proportions of the retained sequences within each sample. No samples were excluded during this filtering process. The affected ASVs and their taxonomic annotations are listed in Supplementary Table S3.

Alpha diversity indices and rarefaction curves were generated using the QIIME 2 and NovoMagic workflows. Observed ASVs and Chao1 described richness, Shannon and Simpson indices described diversity, and the dominance index described community dominance. Good’s coverage was used to estimate the sampling coverage of bacterial communities.

Beta diversity was visualized using principal coordinates analysis (PCoA) based on weighted UniFrac distances in NovoMagic. Group differences were assessed using one-factor permutational multivariate analysis of variance (PERMANOVA) based on Bray–Curtis dissimilarities. For this analysis, ASVs assigned to Cyanobacteria were excluded from the original integer count tables. Retained counts were rarefied without replacement to each dataset’s minimum post-filter depth: 38,220 sequences for laboratory developmental stages, 46,164 for tissue/frass, and 3745 for field larvae. PERMANOVA used 999 unrestricted permutations, and pairwise comparisons were adjusted using Benjamini–Hochberg FDR correction separately within each dataset. Homogeneity of multivariate dispersion was assessed using PERMDISP with spatial medians and 999 permutations. Lingoes correction was applied for dispersion calculations when negative eigenvalues occurred. Overall PERMANOVA and PERMDISP results are provided in Supplementary Table S4, and pairwise PERMANOVA results with FDR correction are provided in Supplementary Table S5. Weighted UniFrac distances were used for ordination, whereas Bray–Curtis dissimilarities were used for PERMANOVA and PERMDISP analyses.

Differences in Shannon and Chao1 indices among groups were assessed in NovoMagic using the Kruskal–Wallis test, followed by Dunn’s post hoc test with FDR correction for multiple comparisons. Significance letters were assigned based on the FDR-adjusted pairwise comparisons. Each pooled sample was treated as one biological replicate and the unit of analysis. Laboratory developmental-stage/adult-sex groups and field instars comprised five biological replicates each, whereas each tissue/frass type comprised four biological replicates. Relative taxon abundances were summarized descriptively and were not subjected to inferential statistical testing.

### 2.5. Prediction of Microbial Functional Potential

ASV abundance tables were uploaded to the PICRUSt v1.1.4 module in NovoMagic for prediction of microbial functional potential. Greengenes v13.5 was used as the reference, and predictions were annotated within the KEGG Orthology (KO) framework and summarized at KEGG level 2. Clustered relative-abundance heatmaps were used for descriptive comparison; no statistical tests were performed for these predicted functional profiles. Sample metadata and individual sequence accessions are provided in Supplementary Table S2 (BioProject PRJNA1530935; SRA study SRP737584). The deposited sequence data are scheduled for public release upon publication.

## 3. Results

### 3.1. Developmental Dynamics and Predicted Functional Potential of the Gut Microbiota in L. sticticalis

**Figure 1:**
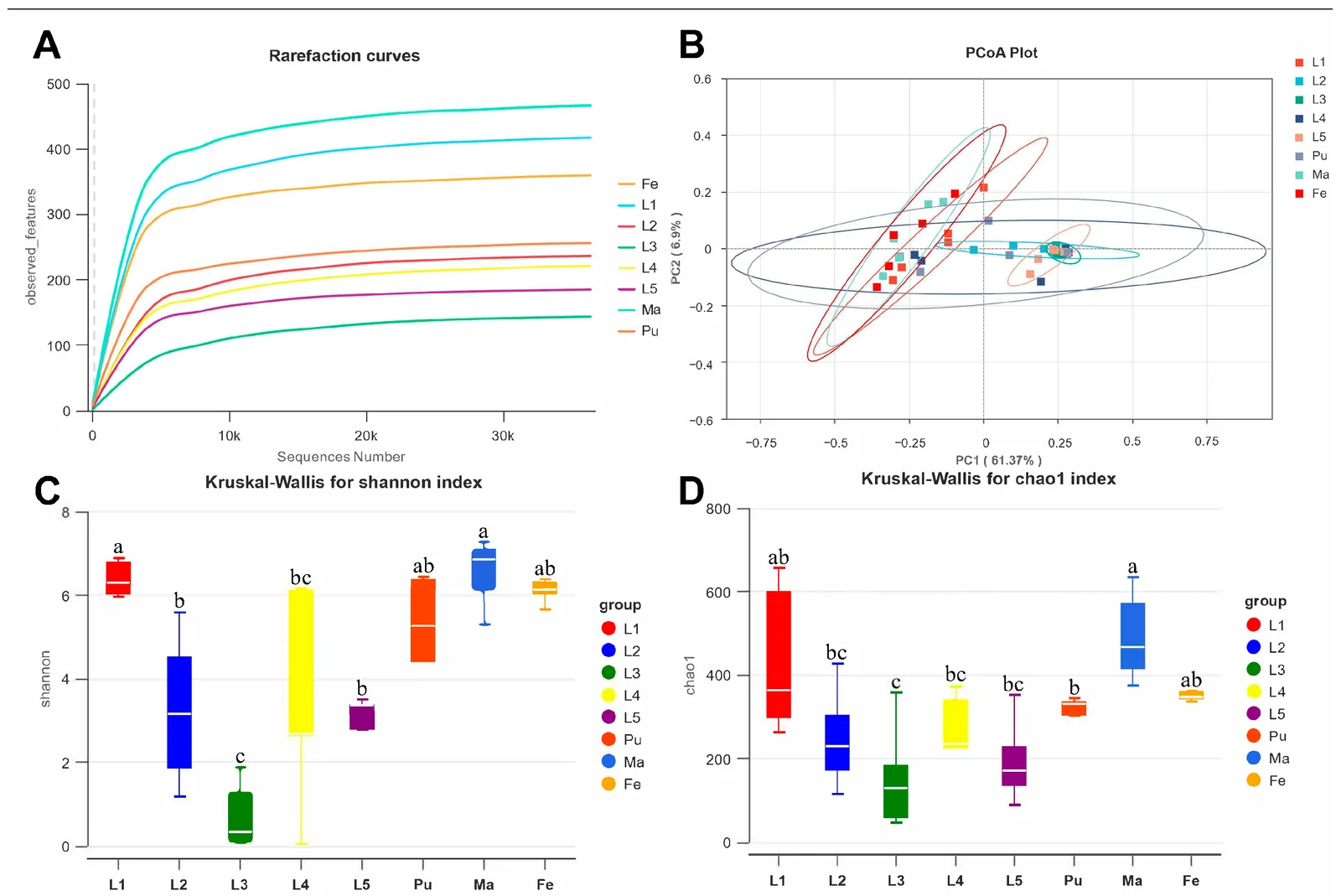
Bacterial community diversity across developmental stages of *L. sticticalis*. (**A**) Rarefaction curves for bacterial communities across developmental stages. (**B**) Principal coordinates analysis (PCoA) of beta diversity based on weighted UniFrac distances. Circles outline the distribution of sample scores within each group. (**C**) Shannon diversity index for first- through fifth-instar larvae (L1–L5), pupae (Pu), female adults (Fe), and male adults (Ma). (**D**) Chao1 richness index for the same developmental stages. Different lowercase letters indicate significant differences among groups (FDR-adjusted *p* < 0.05); groups sharing at least one letter are not significantly different.

A total of 3,493,422 high-quality reads were obtained from gut samples collected across developmental stages of *L. sticticalis*. Rarefaction curves for all samples approached a plateau with increasing sequencing depth, indicating sufficient coverage of most bacterial taxa (Figure 1A).

We analyzed alpha and beta diversity to characterize gut bacterial diversity and community structure across developmental stages of *L. sticticalis*. Alpha diversity differed among stages, as indicated by both the Shannon and Chao1 indices (Figure 1C,D). Shannon diversity was higher in first-instar larvae and adult males than in second- to fifth-instar larvae. Third-instar larvae exhibited the lowest diversity among all groups. The Chao1 index showed a similar pattern of bacterial richness. Beta diversity also revealed pronounced developmental shifts in gut bacterial community structure (Figure 1B). Communities from first-instar larvae clustered closely with those from adult females and males. By contrast, communities from second- to fourth-instar larvae were more similar to pupal communities in the weighted UniFrac ordination. Bray–Curtis PERMANOVA detected overall differences among groups (R^2^ = 0.455, *p* = 0.001), alongside differences in dispersion (PERMDISP *p* = 0.001). Pairwise comparisons distinguished first-instar larvae from male and female adults after FDR correction (Supplementary Table S5).

**Figure 2:**
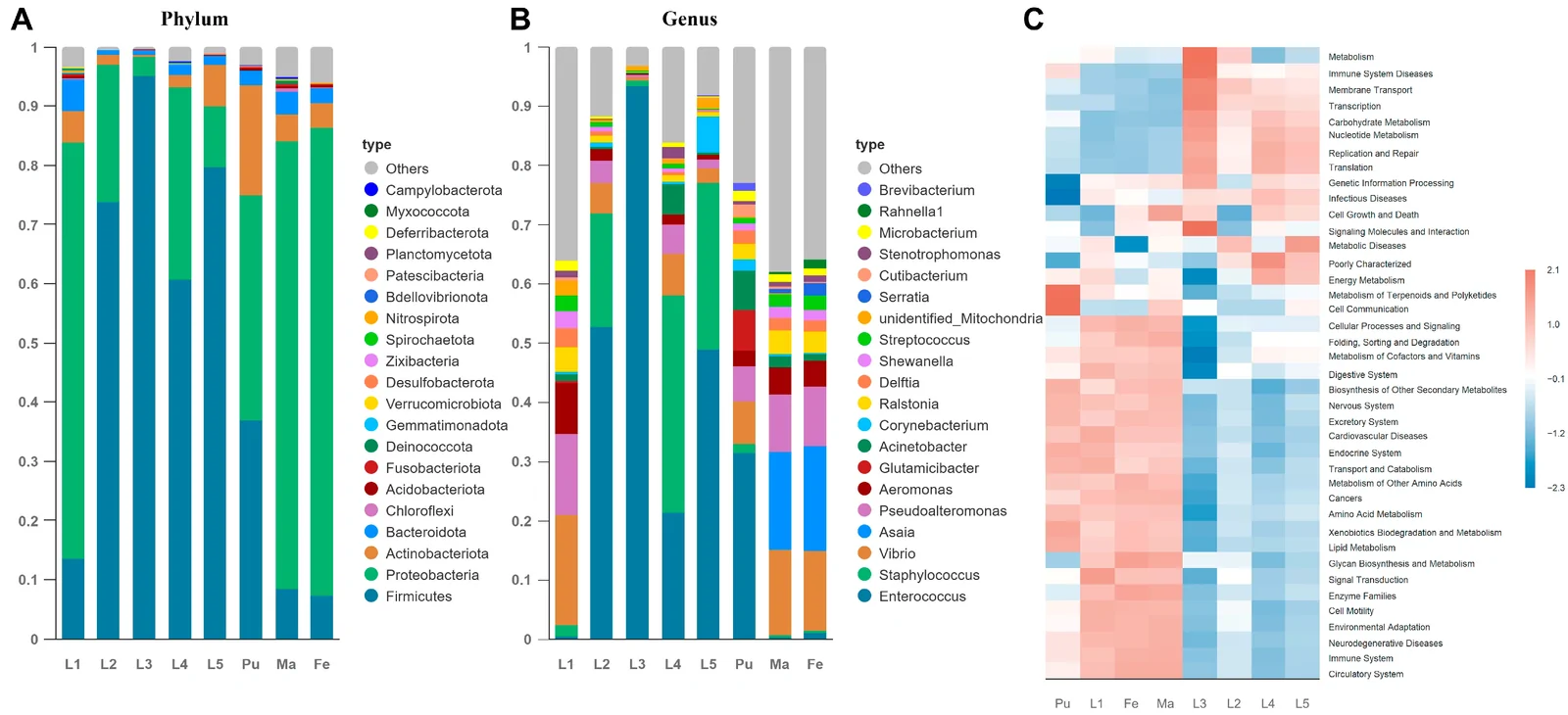
Taxonomic composition and predicted functional profiles of bacterial communities across developmental stages of *L. sticticalis*. (**A**) Bacterial community composition at the phylum level in first- through fifth-instar larvae (L1–L5), pupae (Pu), female adults (Fe), and male adults (Ma). (**B**) Bacterial community composition at the genus level across the same developmental stages. In (**A**,**B**), colors indicate bacterial taxa, “Others” denotes taxa not displayed individually, and columns correspond to sample groups. (**C**) Predicted functional profiles of bacterial communities across developmental stages.

We next examined developmental differences in gut bacterial composition and predicted functional potential. Bacterial communities were analyzed at the phylum and genus levels, followed by PICRUSt-based functional prediction and clustering. Consistent with the diversity patterns, community composition varied markedly across developmental stages at both taxonomic levels. Firmicutes, Proteobacteria, and Actinobacteriota were the dominant phyla throughout development (Figure 2A). Firmicutes predominated in second-, third-, fourth-, and fifth-instar larvae, representing 73.69%, 95.08%, 60.73%, and 79.62% of the community, respectively. Proteobacteria predominated in first-instar larvae and adults. Their relative abundance was 70.35% in first-instar larvae, 79.08% in adult females, and 75.68% in adult males. Actinobacteriota were relatively abundant in fifth-instar larvae (7.10%) and pupae (18.63%).

At the genus level, *Enterococcus* and *Staphylococcus* predominated in second- to fifth-instar larvae and pupae (Figure 2B). *Enterococcus* represented 52.73%, 93.38%, 21.37%, 48.88%, and 31.31% of communities from second-, third-, fourth-, and fifth-instar larvae and pupae, respectively. *Staphylococcus* represented 19.10%, 36.54%, and 28.06% of communities from second-, fourth-, and fifth-instar larvae, respectively. By contrast, *Vibrio* (18.67%) and *Pseudoalteromonas* (13.62%) were the dominant genera in first-instar larvae. Adult communities were dominated by *Asaia*, *Vibrio*, and *Pseudoalteromonas*. These genera represented 17.68%, 13.46%, and 10.12% of female communities and 16.59%, 14.43%, and 9.58% of male communities, respectively.

PICRUSt-based functional prediction revealed stage-dependent variation in the predicted functional potential of the gut microbiota (Figure 2C). Predicted functional profiles in first-instar larvae, pupae, and adults clustered together. These communities showed higher predicted relative abundances of pathways related to lipid and amino acid metabolism, transport and catabolism, as well as KEGG categories annotated as nervous, endocrine, and excretory systems. By contrast, communities from second- to fifth-instar larvae had similar predicted functional profiles. They showed higher predicted relative abundances of pathways related to carbohydrate and nucleotide metabolism, translation, replication and repair, and membrane transport.

### 3.2. Distribution and Predicted Functional Potential of Bacteria Among Tissue and Frass Samples in L. sticticalis

**Figure 3:**
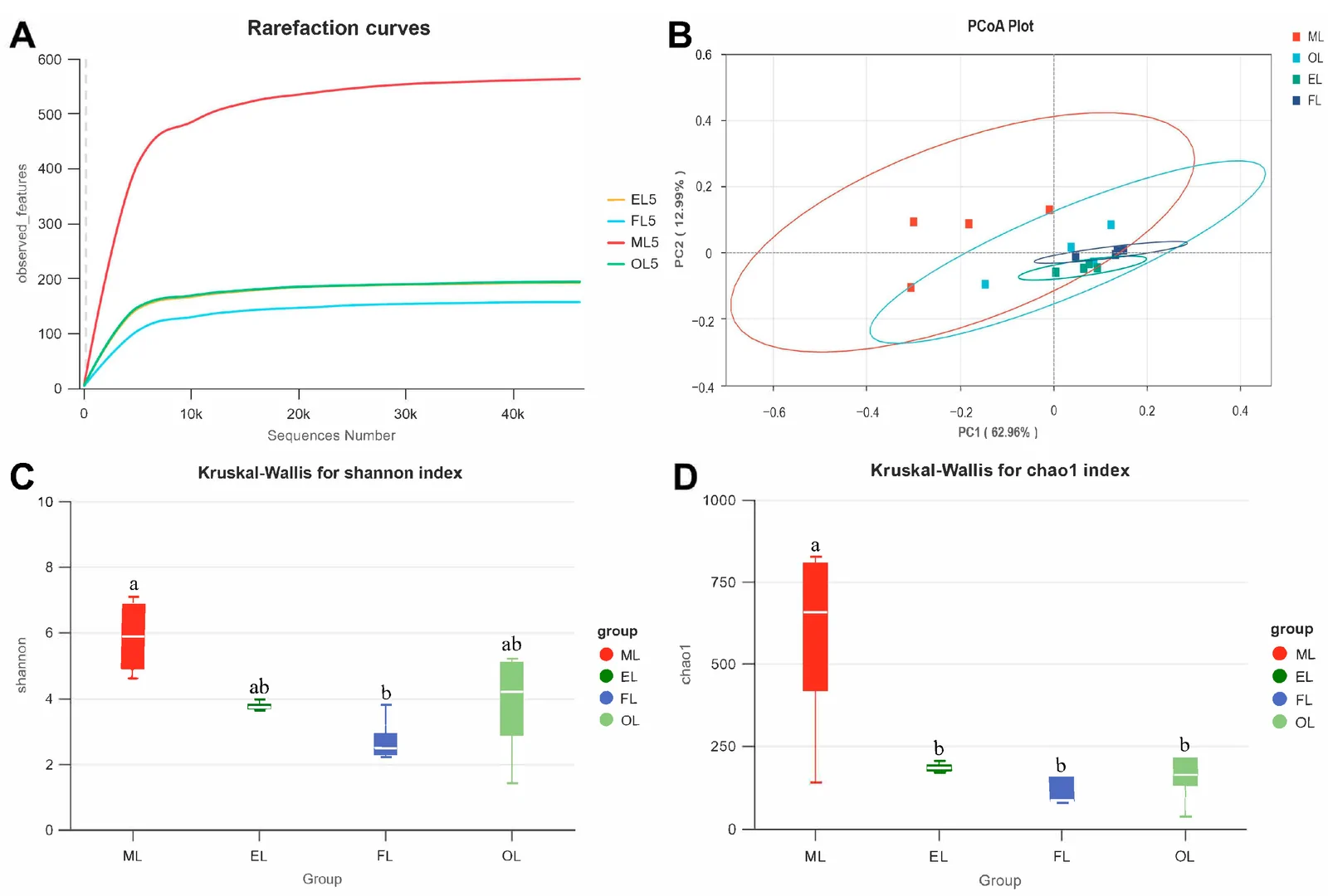
Bacterial community diversity among tissue and frass samples of *L. sticticalis*. (**A**) Rarefaction curves for bacterial communities from different sample types. (**B**) Principal coordinates analysis (PCoA) of beta diversity based on weighted UniFrac distances. Circles outline the distribution of sample scores within each group. (**C**) Shannon diversity index for the midgut (ML), epidermis (EL), frass (FL), and other tissues (OL). (**D**) Chao1 richness index for the same sample types. Different lowercase letters indicate significant differences among groups (FDR-adjusted *p* < 0.05); groups sharing at least one letter are not significantly different.

A total of 1,589,754 high-quality reads were obtained from the midgut, epidermis, frass, and other tissues of *L. sticticalis* larvae. Rarefaction curves for all sample types approached a plateau with increasing sequencing depth, indicating sufficient coverage of most bacterial taxa (Figure 3A).

We analyzed alpha and beta diversity to compare bacterial diversity and community structure across the midgut, epidermis, frass, and other larval tissues. Alpha diversity differed significantly among sample types. The Shannon index was significantly higher in the midgut than in frass (Figure 3C). The Chao1 index was also significantly higher in the midgut than in other tissues (Figure 3D). The weighted UniFrac PCoA showed overlap among sample types (Figure 3B). Bray–Curtis PERMANOVA detected overall differences among sample types (R^2^ = 0.382, *p* = 0.008), although multivariate dispersion also differed (PERMDISP *p* = 0.022). None of the pairwise comparisons remained significant after FDR correction (Supplementary Table S5).

**Figure 4:**
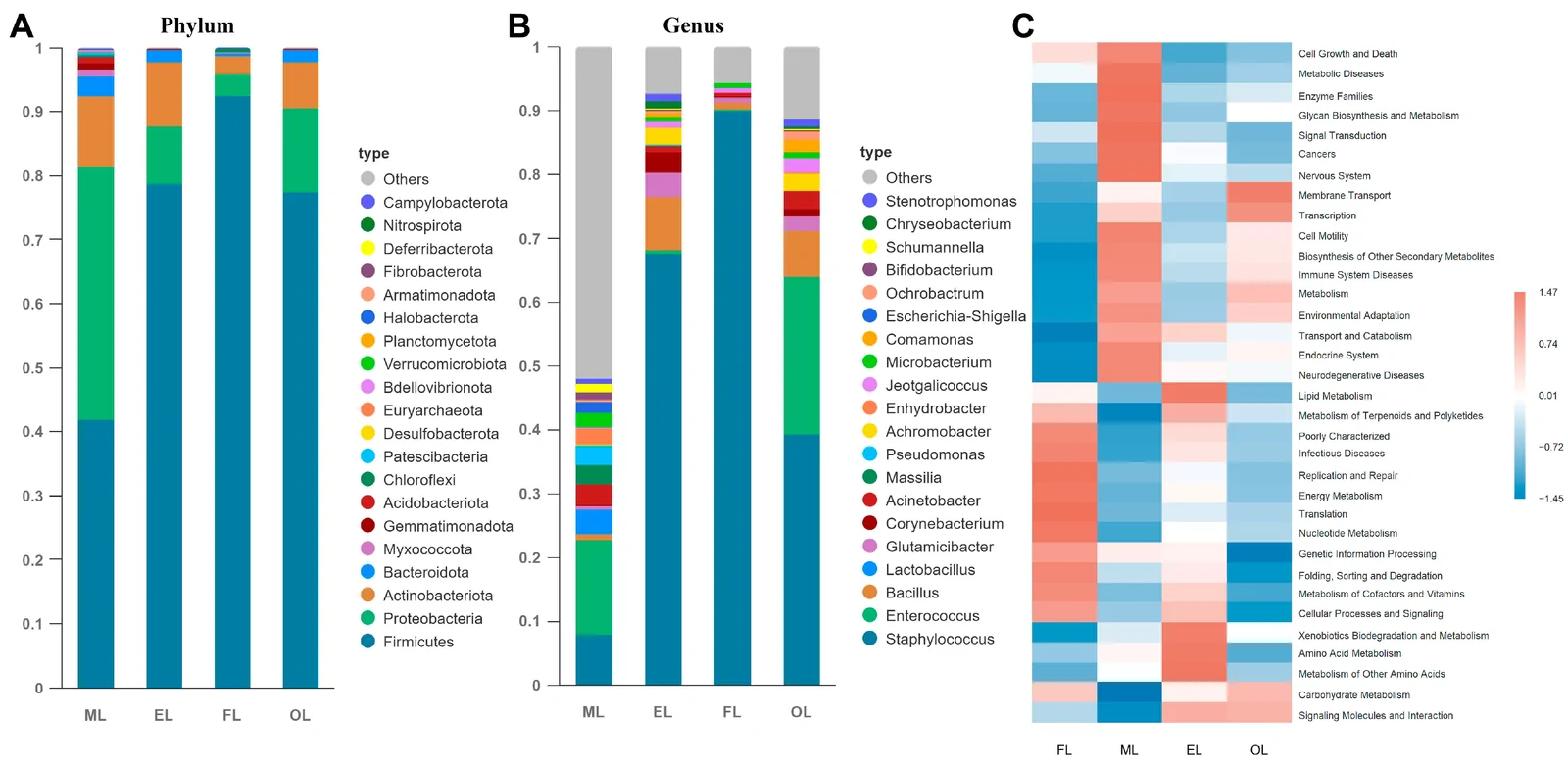
Taxonomic composition and predicted functional profiles of bacterial communities among tissue and frass samples of *L. sticticalis*. (**A**) Bacterial community composition at the phylum level in the midgut (ML), epidermis (EL), frass (FL), and other tissues (OL). (**B**) Bacterial community composition at the genus level across the same sample types. In (**A**,**B**), colors indicate bacterial taxa, “Others” denotes taxa not displayed individually, and columns correspond to sample groups. (**C**) Predicted functional profiles of bacterial communities across sample types.

We next examined sample-associated differences in symbiotic bacterial composition and predicted functional potential. Communities were analyzed at the phylum and genus levels, followed by PICRUSt-based functional prediction and clustering. Firmicutes, Proteobacteria, and Actinobacteriota were the dominant phyla across all sample types (Figure 4A). Firmicutes represented 41.78%, 78.65%, 92.34%, and 77.42% of communities from the midgut, epidermis, frass, and other tissues, respectively. The corresponding proportions were 39.49%, 8.96%, 3.55%, and 13.03% for Proteobacteria and 11.08%, 10.11%, 2.83%, and 7.27% for Actinobacteriota.

At the genus level, *Enterococcus* predominated in the midgut, whereas *Staphylococcus* predominated in the epidermis, frass, and other tissues (Figure 4B). *Enterococcus* represented 24.87% of communities from other tissues, 14.77% from the midgut, 0.42% from the epidermis, and 0.09% from frass. By contrast, *Staphylococcus* represented 7.88% of midgut communities, 67.55% of epidermal communities, 90.01% of frass communities, and 39.09% of other tissue communities.

PICRUSt-based functional prediction also revealed differences among sample types in microbial metabolic potential (Figure 4C). Predicted functional profiles of communities from the midgut and other tissues clustered together. These communities showed higher predicted relative abundances of pathways related to membrane transport, metabolism, and transport and catabolism, as well as KEGG categories annotated as immune, endocrine, excretory, and nervous systems. By contrast, epidermal and frass communities formed a separate cluster. These communities showed higher predicted relative abundances of pathways related to cellular processes and signaling, translation, and replication and repair, as well as KEGG categories annotated as infectious diseases.

### 3.3. Dynamics and Predicted Functional Potential of the Gut Microbiota in Wild L. sticticalis

**Figure 5:**
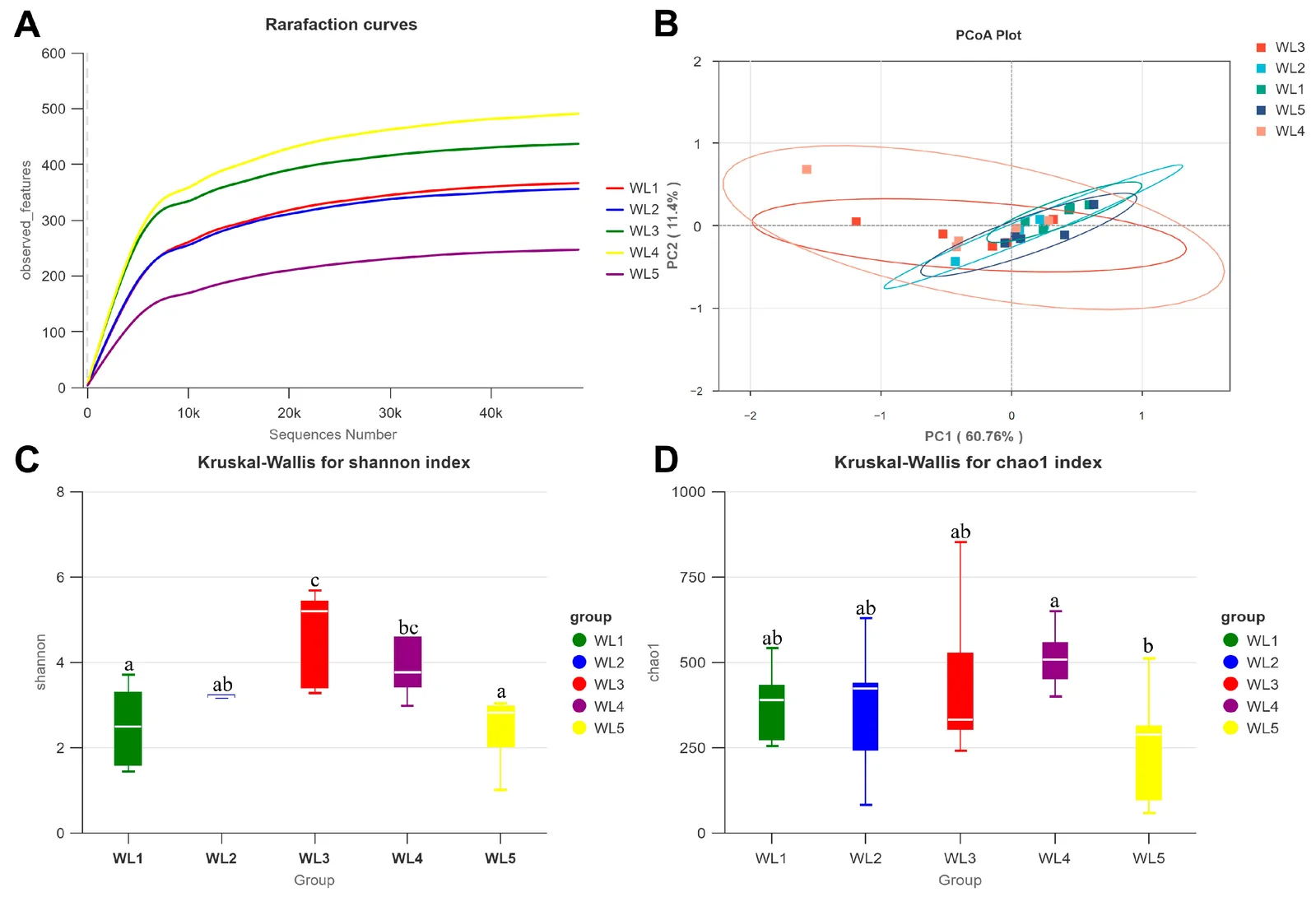
Bacterial community diversity across larval instars of wild *L. sticticalis*. (**A**) Rarefaction curves for bacterial communities across larval instars. (**B**) Principal coordinates analysis (PCoA) of beta diversity based on weighted UniFrac distances. Circles outline the distribution of sample scores within each group. (**C**) Shannon diversity index for first- through fifth-instar larvae of wild *L. sticticalis* (WL1–WL5). (**D**) Chao1 richness index for the same larval instars. Different lowercase letters indicate significant differences among groups (FDR-adjusted *p* < 0.05); groups sharing at least one letter are not significantly different.

A total of 2,050,195 high-quality reads were obtained from wild larval gut samples, and the company-delivered ASV table contained 1,686,490 sequences. Rarefaction curves for all samples approached a plateau with increasing sequencing depth, indicating sufficient coverage of most bacterial taxa (Figure 5A).

We analyzed alpha and beta diversity to characterize changes in the gut microbiota during larval development in wild *L. sticticalis*. Gut bacterial alpha diversity differed among instars, as indicated by both the Shannon and Chao1 indices (Figure 5C,D). Shannon diversity was highest in third-instar larvae. First-instar larvae had the lowest Shannon diversity. By contrast, the Chao1 index increased gradually from the first to fourth instar and then declined markedly at the fifth instar. The weighted UniFrac PCoA showed no clear separation across instars (Figure 5B). Bray–Curtis PERMANOVA detected no significant overall difference among instars (R^2^ = 0.143, *p* = 0.669). Multivariate dispersion also did not differ significantly (PERMDISP *p* = 0.920; Supplementary Table S4).

**Figure 6:**
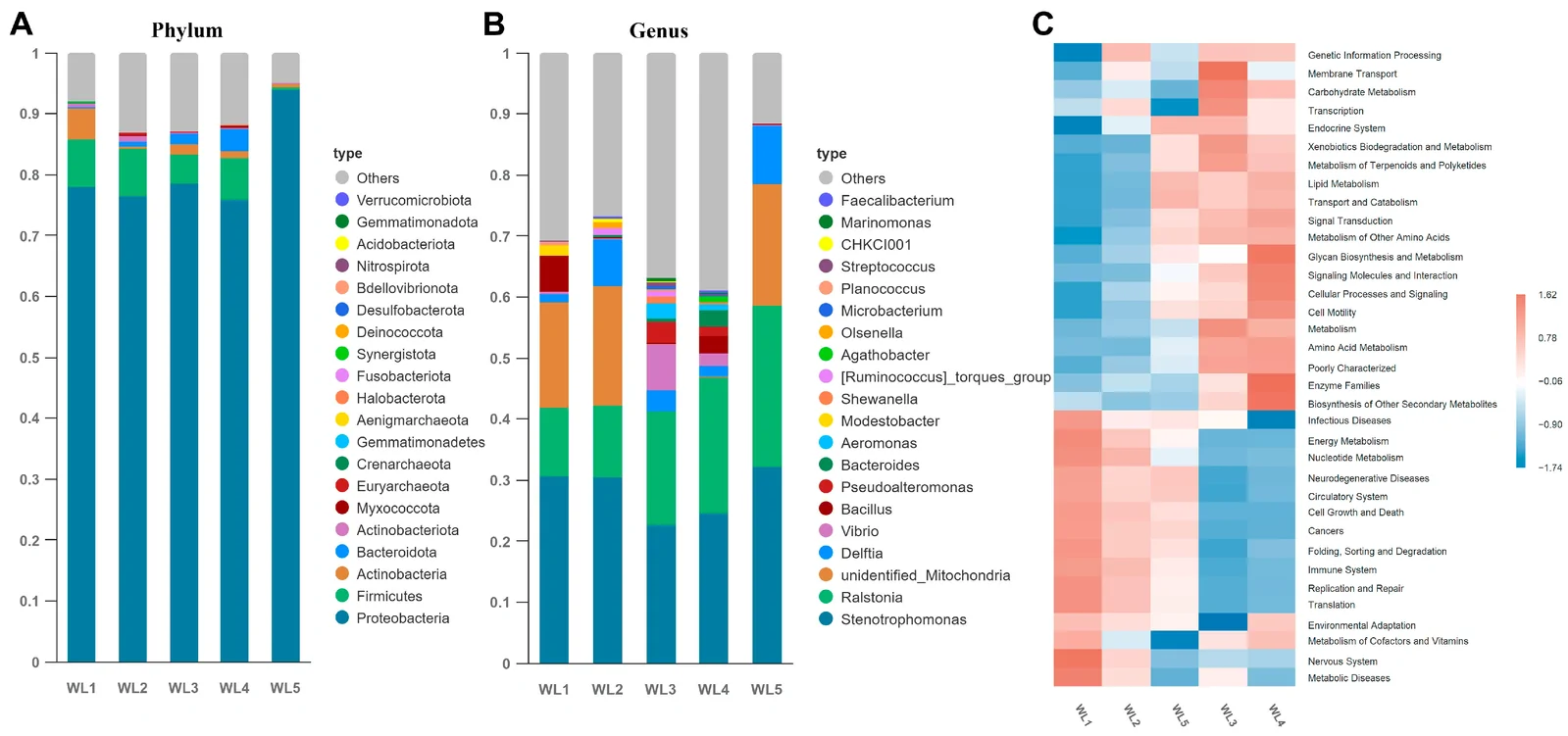
Taxonomic composition and predicted functional profiles of bacterial communities across larval instars of wild *L. sticticalis*. (**A**) Bacterial community composition at the phylum level in first- through fifth-instar larvae (WL1–WL5). (**B**) Bacterial community composition at the genus level across the same larval instars. In (**A**,**B**), colors indicate bacterial taxa, “Others” denotes taxa not displayed individually, and columns correspond to sample groups. (**C**) Predicted functional profiles of bacterial communities across larval instars.

We next examined differences in gut bacterial composition and predicted functional potential among wild *L. sticticalis* larval instars. Community structure was analyzed at the phylum and genus levels, followed by PICRUSt-based functional prediction and clustering. Proteobacteria dominated the gut microbiota from the first through fifth instars, with Firmicutes as the second most abundant phylum. Actinobacteriota, Bacteroidota, Myxococcota, and Crenarchaeota were consistently detected at low relative abundances throughout larval development (Figure 6A). Proteobacteria represented 77.89%, 76.50%, 78.45%, and 75.94% of communities from first- to fourth-instar larvae, respectively. Their relative abundance was highest in fifth-instar larvae (93.91%). By contrast, Firmicutes represented only 0.39% of communities from fifth-instar larvae. This proportion was markedly lower than those in first- to fourth-instar larvae (7.92%, 7.64%, 4.85%, and 6.84%, respectively).

At the genus level, *Stenotrophomonas* was dominant across all five instars of wild larvae, whereas *Ralstonia* was the second most abundant genus (Figure 6B). *Vibrio*, *Delftia*, *Bacillus*, *Bacteroides*, and several other genera were consistently detected at low abundances in all instars. *Stenotrophomonas* abundance was lowest in third-instar larvae (22.64%) and highest in fifth-instar larvae (32.22%). *Ralstonia* relative abundance increased progressively across the first through fifth larval instars, reaching 11.26%, 11.79%, 18.57%, 22.13%, and 26.33%, respectively.

PICRUSt-based functional prediction revealed differences in gut microbial metabolic potential among instars of wild *L. sticticalis* (Figure 6C). Predicted functional profiles in first- and second-instar larvae clustered together. These communities showed higher predicted relative abundances of pathways related to genetic information processing and fundamental cellular metabolism. The more represented predicted categories included energy and nucleotide metabolism, translation, replication and repair, and KEGG categories annotated as immune system functions. The predicted relative abundances of these categories tended to decline as larvae developed. By contrast, pathways related to carbohydrate, amino acid, and lipid metabolism showed higher predicted relative abundances in the middle and late instars. Glycan biosynthesis and metabolism, signal transduction, environmental adaptation, cell motility, and transport and catabolism showed similar patterns. These predictions suggest a developmental shift in the predicted functional profile of the gut microbiota from basic cellular processes toward nutrient metabolism and environmental adaptation.

## 4. Discussion

In *L. sticticalis*, bacterial composition varied across development in the laboratory population, while diversity and key genera differed among tissue and frass samples. Laboratory and wild populations differed in community composition and predicted functions. Wild larvae were dominated by *Stenotrophomonas* and *Ralstonia*, whereas *Enterococcus* and *Staphylococcus* were more abundant under laboratory conditions. These findings identify candidate bacterial taxa for studies of host adaptation and pest management.

The insect gut microbiota changes dynamically across developmental stages [12,13,37]. In *L. sticticalis*, Firmicutes predominated in second- to fifth-instar larvae, whereas Proteobacteria dominated first-instar larvae and adults (Figure 2), consistent with studies of *L. sticticalis* and other lepidopterans [15,35,38]. Larvae consume polysaccharide-rich leaves, and many gut bacteria can degrade carbohydrates [39]. During pupation, midgut remodeling and host metabolic changes may alter conditions for microbial colonization [40,41]. Overall, these shifts may reflect adaptation of the gut microbiota to stage-specific dietary, nutritional, and metabolic demands.

At the genus level, *Enterococcus* predominated in second- to fifth-instar larvae and pupae, whereas *Vibrio* and *Pseudoalteromonas* dominated first-instar larvae and adults. *Enterococcus* may contribute to digestion, microbial competition, and host defense [42,43,44,45]. Some strains exhibit proteolytic activity [44]. In *Spodoptera littoralis*, *Enterococcus mundtii* produces the bacteriocin mundticin KS, which inhibits invading bacteria and contributes to the regulation of gut microbial communities [45].

A similar dominance pattern has been reported in *Etiella zinckenella* [46]. Antibacterial and microbiota-modulatory effects have been reported for *Pseudoalteromonas ruthenica* in fish [47], suggesting candidate activities for investigation in insects [48]. *Asaia* was also abundant in adults and has been reported in *Pieris rapae*, mosquitoes, and *Sogatella furcifera* [49,50]. Its association with sugar-feeding insects [50,51] suggests that its abundance may be related to the honey solution provided during rearing.

Alpha diversity was lowest in third-instar larvae of *L. sticticalis* and higher in adults, with adult males showing the highest bacterial richness (Figure 1C,D). The insect immune system can shape the gut microbiota by regulating microbial density and community composition [10,52,53,54]. Thus, this pattern may partly reflect immune-mediated filtering during the third instar, alongside differences in diet, gut morphology, and physiology [30,52]. Higher bacterial richness in adults may also reflect greater physiological demands, including potential microbial roles in reproduction [38].

Beta diversity analysis showed that the gut microbiota of first-instar larvae partly resembled that of adults. Functional predictions likewise indicated broadly similar profiles in these stages. Together, these findings suggest a possible link between adult- and offspring-associated bacterial communities. Similar patterns have also been reported in other lepidopterans [55,56]. Further studies could distinguish vertical transmission from bacterial acquisition through food or the environment.

Microbial diversity and community composition differed among tissue and frass samples of *L. sticticalis* larvae [25,57,58]. The midgut, epidermis, frass, and other tissues nevertheless shared the dominant phyla, resembling phylum-level patterns in *Spodoptera frugiperda* and *Bombyx mori* [59,60]. *Enterococcus* predominated in the midgut, whereas *Staphylococcus* was most abundant in epidermal and frass samples. Some *Enterococcus* strains have been associated with digestion, antimicrobial activity, metamorphic development, and insecticide detoxification [44,45,61,62]. For example, *E. casseliflavus* EMBL-3 has been reported to degrade chlorantraniliprole and increase resistance in *S. frugiperda* [62]. In insect models, some *Staphylococcus* strains persist within hosts or cause disease [63,64], suggesting that host colonization and potential pathogenicity warrant further investigation in *L. sticticalis*. Protective effects of cuticular *Pantoea* have also been demonstrated in *Grapholita molesta* [23], providing a basis for exploring possible defensive roles of epidermis-associated bacteria.

Compared with the laboratory population, wild *L. sticticalis* larvae harbored distinct gut bacterial communities, with substantial overlap across field instars. These differences may reflect diet, population origin, sampling time, temperature, and moisture conditions [30,36,65,66,67]. Temperature-associated changes in gut bacterial composition have been reported in *L. sticticalis* and *S. exigua* [36,66]. Gut microbiota also differed between dry and rainy seasons in *S. frugiperda* [67], while studies of Arctic insects linked bacterial diversity to temperature and humidity [65].

*Stenotrophomonas* and *Ralstonia* dominated wild larvae but were markedly less abundant in the laboratory population. In *Spodoptera frugiperda*, gut enrichment of *Stenotrophomonas* has been associated with increased insecticide resistance, and strains of this genus can efficiently degrade beta-cypermethrin [68]. Thus, the high abundance of *Stenotrophomonas* in wild *L. sticticalis* identifies this genus as a candidate for investigating insecticide-associated processes.

*Ralstonia* abundance increased gradually during wild larval development. In *Dorcus hopei hopei*, high abundance of this genus has been associated with enriched predicted functions for xenobiotic biodegradation and metabolism [69]. This association suggests a possible role in detoxification. *Ralstonia* is also a dominant gut bacterium in *Tyria jacobaeae* and appears not to originate entirely from soil [70]. These observations identify xenobiotic metabolism as a candidate function for investigation; the source and functional role of *Ralstonia* in *L. sticticalis* remain unresolved.

## 5. Conclusions

The bacterial communities associated with *Loxostege sticticalis* exhibited dynamic variation across developmental stages, tissue and frass samples, and laboratory and field conditions. Host development and environmental conditions were associated with microbiota variation, with distinct community patterns between laboratory-reared and field populations. Differences among tissue and frass samples further revealed the spatial heterogeneity of host-associated bacterial communities, highlighting the importance of ecological context in understanding insect–microbiota interactions. Together, these findings improve our understanding of bacterial community variation in *L. sticticalis* and identify several microbial taxa and predicted functions for further investigation. Further studies are needed to clarify the ecological and physiological roles of these bacteria and their relationships with host development and environmental conditions.

## Data Availability

The raw sequencing data generated in this study have been deposited in the NCBI Sequence Read Archive (SRA) under BioProject accession number https://www.ncbi.nlm.nih.gov/bioproject/PRJNA1530935 (accessed on 17 September 2026).

## Supplementary Materials

The following supporting information can be downloaded at: https://www.mdpi.com/article/10.3390/insects17101068/s1, Table S1. Per-sample sequencing depth and taxonomic filtering; Table S2. Pooled-sample metadata and SRA accession mapping; Table S3. Cyanobacteria-assigned ASVs excluded by taxonomic filtering; Table S4. Overall Bray–Curtis PERMANOVA and PERMDISP results; Table S5. Pairwise Bray–Curtis PERMANOVA with FDR correction.

## Abbreviations

The following abbreviations are used in this manuscript:

16S rRNA	16S ribosomal RNA
ASV	amplicon sequence variant
CTAB	cetyltrimethylammonium bromide
DADA2	Divisive Amplicon Denoising Algorithm 2
PCoA	principal coordinates analysis
PCR	polymerase chain reaction
PICRUSt	Phylogenetic Investigation of Communities by Reconstruction of Unobserved States
QIIME 2	Quantitative Insights Into Microbial Ecology 2
L1–L5	first- to fifth-instar larvae of the laboratory population
WL1–WL5	first- to fifth-instar larvae of the wild population
Pu	pupae
Fe	female adults
Ma	male adults
ML	midgut samples
EL	epidermal samples
FL	frass samples
OL	other tissue samples
