# Supplementary material for: Bacterial Community Structural Dynamics and Predicted Functional Potential in Loxostege sticticalis
Source: Insects. 2026 Oct 15;17(10):1068. doi: 10.3390/insects17101068 (PMC13705498; doi:10.3390/insects17101068)
Supplement: Supplementary file 1 [file insects-17-01068-s001.zip › insects-4604625-supplementary.pdf]

Table S1. Per-sample sequencing depth and taxonomic filtering

Manuscript sample ID	Company ASV sample ID	Dataset	Developmental stage	Sample material	Biological replicate	Raw paired-end read pairs	Merged sequences	Quality-filtered merged sequences	Non-chimeric sequences	Company ASV counts before rarefaction	Company-delivered rarefaction depth	Cyanobacteria sequences removed	Post-filter sequences	Bray-Curtis PERMANOVA rarefaction depth	Included in manuscript analysis
L1.1	SL1.1	lab	First-instar larva	Midgut	1	88450	87277	85762	85241	78985	58529	39605	39380	38220	TRUE
L1.2	SL1.2	lab	First-instar larva	Midgut	2	95448	90216	88542	87870	79888	58529	8342	71546	38220	TRUE
L1.3	SL1.3	lab	First-instar larva	Midgut	3	92735	89137	87106	86543	79613	58529	41393	38220	38220	TRUE
L1.4	SL1.4	lab	First-instar larva	Midgut	4	93596	87647	86335	85200	77780	58529	7065	70715	38220	TRUE
L1.5	SL1.5	lab	First-instar larva	Midgut	5	88016	86685	85006	84310	78350	58529	4570	73780	38220	TRUE
L2.1	SL2.1	lab	Second-instar larva	Midgut	1	89931	89286	87431	84939	76923	58529	3561	73362	38220	TRUE
L2.2	SL2.2	lab	Second-instar larva	Midgut	2	89289	88107	86404	86080	79184	58529	415	78769	38220	TRUE
L2.3	SL2.3	lab	Second-instar larva	Midgut	3	88664	88009	86288	84753	77177	58529	640	76537	38220	TRUE
L2.4	SL2.4	lab	Second-instar larva	Midgut	4	99381	97364	95391	94631	86766	58529	2745	84021	38220	TRUE
L2.5	SL2.5	lab	Second-instar larva	Midgut	5	88649	87746	85811	84831	78229	58529	2259	75970	38220	TRUE
L3.1	SL3.1	lab	Third-instar larva	Midgut	1	87246	86384	84716	84250	78662	58529	37499	41163	38220	TRUE
L3.2	SL3.2	lab	Third-instar larva	Midgut	2	89033	88326	86830	86693	81288	58529	19179	62109	38220	TRUE
L3.3	SL3.3	lab	Third-instar larva	Midgut	3	85819	84850	82883	82484	75384	58529	73	75311	38220	TRUE
L3.4	SL3.4	lab	Third-instar larva	Midgut	4	88118	87070	85326	85032	78731	58529	9555	69176	38220	TRUE
L3.5	SL3.5	lab	Third-instar larva	Midgut	5	92530	91859	90049	89791	83257	58529	299	82958	38220	TRUE
L4.1	SL4.1	lab	Fourth-instar larva	Midgut	1	93252	87138	85775	85484	79750	58529	1121	78629	38220	TRUE
L4.2	SL4.2	lab	Fourth-instar larva	Midgut	2	92093	91487	89920	89471	83600	58529	1902	81698	38220	TRUE
L4.3	SL4.3	lab	Fourth-instar larva	Midgut	3	72898	67971	66063	64829	58529	58529	2506	56023	38220	TRUE
L4.4	SL4.4	lab	Fourth-instar larva	Midgut	4	94704	88415	86579	85636	79771	58529	8689	71082	38220	TRUE
L4.5	SL4.5	lab	Fourth-instar larva	Midgut	5	87400	86952	85458	85134	79523	58529	4256	75267	38220	TRUE
L5.1	SL5.1	lab	Fifth-instar larva	Midgut	1	100837	94424	92694	92494	85898	58529	1782	84116	38220	TRUE
L5.2	SL5.2	lab	Fifth-instar larva	Midgut	2	92522	89066	87327	86862	80560	58529	5829	74731	38220	TRUE
L5.3	SL5.3	lab	Fifth-instar larva	Midgut	3	87466	86319	83300	80406	72787	58529	30711	42076	38220	TRUE
L5.4	SL5.4	lab	Fifth-instar larva	Midgut	4	91872	89227	87621	87257	81594	58529	335	81259	38220	TRUE
L5.5	SL5.5	lab	Fifth-instar larva	Midgut	5	96553	95285	93703	93568	87594	58529	12098	75496	38220	TRUE

Pu.1	P1	lab	Pupa	Midgut	1	90405	87621	86044	85401	79557	58529	90	79467	38220	TRUE
Pu.2	P2	lab	Pupa	Midgut	2	94121	87286	85884	82741	76777	58529	16	76761	38220	TRUE
Pu.3	P3	lab	Pupa	Midgut	3	105490	99652	97851	96410	90012	58529	0	90012	38220	TRUE
Pu.4	P4	lab	Pupa	Midgut	4	91409	89462	88023	87291	81556	58529	54	81502	38220	TRUE
Pu.5	P5	lab	Pupa	Midgut	5	94506	93757	91892	91385	84800	58529	9	84791	38220	TRUE
Ma.1	M1	lab	Male adult	Midgut	1	95182	88929	87295	86742	78618	58529	418	78200	38220	TRUE
Ma.2	M2	lab	Male adult	Midgut	2	101009	94676	93003	90824	83716	58529	464	83252	38220	TRUE
Ma.3	M3	lab	Male adult	Midgut	3	91086	88502	86837	86458	79465	58529	1812	77653	38220	TRUE
Ma.4	M4	lab	Male adult	Midgut	4	91563	87129	85707	85084	78905	58529	484	78421	38220	TRUE
Ma.5	M5	lab	Male adult	Midgut	5	101638	96235	94530	91611	84213	58529	282	83931	38220	TRUE
Fe.1	F1	lab	Female adult	Midgut	1	96404	87452	85924	83376	77394	58529	987	76407	38220	TRUE
Fe.2	F2	lab	Female adult	Midgut	2	91195	87674	86105	84875	77656	58529	1963	75693	38220	TRUE
Fe.3	F3	lab	Female adult	Midgut	3	101961	92130	90203	89880	80847	58529	346	80501	38220	TRUE
Fe.4	F4	lab	Female adult	Midgut	4	94165	87441	85990	85655	79893	58529	92	79801	38220	TRUE
Fe.5	F5	lab	Female adult	Midgut	5	92867	87522	85814	82962	74950	58529	174	74776	38220	TRUE
EL5.1	NE1	tissue	Fifth-instar larva	Epidermis	1	107252	106450	104912	104156	99670	61501	1218	98452	46164	TRUE
EL5.2	NE2	tissue	Fifth-instar larva	Epidermis	2	65938	65342	64422	63971	61501	61501	101	61400	46164	TRUE
EL5.3	NE3	tissue	Fifth-instar larva	Epidermis	3	75107	74675	73685	72990	70036	61501	150	69886	46164	TRUE
EL5.4	NE4	tissue	Fifth-instar larva	Epidermis	4	104748	104070	102608	101949	97806	61501	462	97344	46164	TRUE
FL5.1	NF1	tissue	Fifth-instar larva	Frass	1	102769	102134	100766	99161	93727	61501	47563	46164	46164	TRUE
FL5.2	NF2	tissue	Fifth-instar larva	Frass	2	103636	103108	101679	100812	96651	61501	24438	72213	46164	TRUE
FL5.3	NF3	tissue	Fifth-instar larva	Frass	3	105538	104819	103201	98285	91474	61501	9138	82336	46164	TRUE
FL5.4	NF4	tissue	Fifth-instar larva	Frass	4	106772	106238	104867	103533	98481	61501	23795	74686	46164	TRUE
ML5.1	NM1	tissue	Fifth-instar larva	Midgut	1	102232	101624	100326	98057	93926	61501	12795	81131	46164	TRUE
ML5.2	NM2	tissue	Fifth-instar larva	Midgut	2	118140	117237	115636	112848	107551	61501	4772	102779	46164	TRUE
ML5.3	NM3	tissue	Fifth-instar larva	Midgut	3	113876	113197	111700	110108	105750	61501	92	105658	46164	TRUE
ML5.4	NM4	tissue	Fifth-instar larva	Midgut	4	102570	102148	100838	100417	96948	61501	6	96942	46164	TRUE
OL5.1	NO1	tissue	Fifth-instar larva	Other internal tissues	1	106303	104247	102735	100966	97071	61501	933	96138	46164	TRUE
OL5.2	NO2	tissue	Fifth-instar larva	Other internal tissues	2	102454	101610	100202	99531	95678	61501	115	95563	46164	TRUE
OL5.3	NO3	tissue	Fifth-instar larva	Other internal tissues	3	104678	103774	102108	100846	95514	61501	560	94954	46164	TRUE
OL5.4	NO4	tissue	Fifth-instar larva	Other internal tissues	4	102700	101838	100069	99700	95291	61501	173	95118	46164	TRUE
WL1.1	WL2.1	wild	First-instar larva	Midgut	1	99807	94962	93427	91394	86709	48652	70025	16684	3745	TRUE
WL1.2	WL2.2	wild	First-instar larva	Midgut	2	95907	83025	81438	68659	63552	48652	3377	60175	3745	TRUE
WL1.3	WL2.3	wild	First-instar larva	Midgut	3	94798	87427	85723	82338	77099	48652	48488	28611	3745	TRUE
WL1.4	WL2.4	wild	First-instar larva	Midgut	4	89848	77656	75521	74623	69274	48652	55338	13936	3745	TRUE
WL1.5	WL2.5	wild	First-instar larva	Midgut	5	100912	87859	86257	79363	72645	48652	24132	48513	3745	TRUE
WL2.1	WL3.1	wild	Second-instar larva	Midgut	1	72153	65233	63515	62953	58392	48652	54647	3745	3745	TRUE
WL2.2	WL3.2	wild	Second-instar larva	Midgut	2	95169	83238	81551	74936	68058	48652	30497	37561	3745	TRUE

WL2.3	WL3.3	wild	Second-instar larva	Midgut	3	74824	59274	58266	53514	48652	48652	94	48558	3745	TRUE
WL2.4	WL3.4	wild	Second-instar larva	Midgut	4	89399	77808	76469	60960	53936	48652	97	53839	3745	TRUE
WL2.5	WL3.5	wild	Second-instar larva	Midgut	5	100980	87798	86271	75764	67150	48652	17544	49606	3745	TRUE
WL3.1	WL4.1	wild	Third-instar larva	Midgut	1	109105	93505	91968	85079	78386	48652	1568	76818	3745	TRUE
WL3.2	WL4.2	wild	Third-instar larva	Midgut	2	76751	65698	64744	62546	55406	48652	28	55378	3745	TRUE
WL3.3	WL4.3	wild	Third-instar larva	Midgut	3	96098	88736	87141	72276	64832	48652	33	64799	3745	TRUE
WL3.4	WL4.4	wild	Third-instar larva	Midgut	4	100303	93834	92114	88949	79862	48652	45	79817	3745	TRUE
WL3.5	WL4.5	wild	Third-instar larva	Midgut	5	99260	87558	86438	75917	68164	48652	13	68151	3745	TRUE
WL4.1	WL5.1	wild	Fourth-instar larva	Midgut	1	103563	87604	85570	79975	73785	48652	28881	44904	3745	TRUE
WL4.2	WL5.2	wild	Fourth-instar larva	Midgut	2	91943	79715	78321	71709	57529	48652	166	57363	3745	TRUE
WL4.3	WL5.3	wild	Fourth-instar larva	Midgut	3	94787	85300	84005	71052	62230	48652	247	61983	3745	TRUE
WL4.4	WL5.4	wild	Fourth-instar larva	Midgut	4	102978	91635	89997	81350	75808	48652	210	75598	3745	TRUE
WL4.5	WL5.5	wild	Fourth-instar larva	Midgut	5	91868	80070	78178	76720	54956	48652	15	54941	3745	TRUE
WL5.1	WL6.1	wild	Fifth-instar larva	Midgut	1	97514	88503	86966	78511	72558	48652	413	72145	3745	TRUE
WL5.2	WL6.2	wild	Fifth-instar larva	Midgut	2	93434	81103	79118	70958	61545	48652	89	61456	3745	TRUE
WL5.3	WL6.3	wild	Fifth-instar larva	Midgut	3	92826	90600	88308	87067	80364	48652	64757	15607	3745	TRUE
WL5.4	WL6.4	wild	Fifth-instar larva	Midgut	4	98948	85725	84157	74974	66233	48652	176	66057	3745	TRUE
WL5.5	WL6.5	wild	Fifth-instar larva	Midgut	5	92908	86758	84732	77347	69365	48652	697	68668	3745	TRUE

Notes

**Sample coverage**	The manuscript includes 81 pooled samples: 40 laboratory developmental-stage/adult-sex samples, 16 fifth-instar tissue/frass samples, and 25 field larval samples. Four additional DM samples from the tissue sequencing project are outside the manuscript scope.
**Analysis unit**	Each sample is one pooled biological replicate and one unit of analysis. Laboratory developmental-stage/adult-sex groups and field instars each have five pooled biological replicates. Each tissue/frass type has four pooled biological replicates. Individuals within a pool are not counted as separate replicates.
**Pool size**	For laboratory developmental-stage/adult-sex and field-instar samples, each replicate contains midguts from 5–15 individuals. Pool size varies with body size to obtain sufficient material and DNA. For the fifth-instar tissue comparison, each midgut, epidermis or other-internal-tissue replicate pools the corresponding material from five larvae. Each frass replicate pools frass from ten larvae. Different sample types originate from different insects.
**Female lineages**	Five independent female lineages were established for the laboratory developmental-stage/adult-sex comparison. Each group contains one pooled sample from each lineage. Individuals within a pool originate from the same female lineage, and the five pools within a group originate from five different female lineages.
**Read counts**	Raw counts are paired-end read pairs. Merged, quality-filtered and non-chimeric counts are sequences at the corresponding quality-control steps. ASV counts are counts in the company ASV table before rarefaction. These processing-stage counts are not interchangeable.
**Initial rarefaction**	Company-delivered rarefaction depths describe the initial project analyses. They do not specify the depths used for the later filtered cloud alpha-diversity figures.
**Bray–Curtis rarefaction**	Bray–Curtis analyses use the original integer ASV counts after exclusion of Cyanobacteria-assigned ASVs. Counts were rarefied once without replacement to 38,220 (lab), 46,164 (tissue), or 3,745 (wild) sequences per pooled sample. PERMANOVA used one factor, 999 unrestricted permutations, and scikit-bio v0.7.4.
**Source records**	Company sequencing reports, ASV count tables and the existing filtered Bray–Curtis analysis records generated on 5 October 2026.

Table S2. Pooled-sample metadata and SRA accession mapping

Manuscript sample ID	Submitted sample name	Company ASV sample ID	Population	Developmental stage	Sample material	Biological replicate	SRA run accession	BioSample accession	SRA study accession	BioProject accession	Individuals per pooled replicate
L1.1	SL1.1	SL1.1	Laboratory-reared	First-instar larva	Midgut	1	SRR40720519	SAMN63217852	SRP737584	PRJNA1530935	5–15
L1.2	SL1.2	SL1.2	Laboratory-reared	First-instar larva	Midgut	2	SRR40720518	SAMN63217853	SRP737584	PRJNA1530935	5–15
L1.3	SL1.3	SL1.3	Laboratory-reared	First-instar larva	Midgut	3	SRR40720507	SAMN63217854	SRP737584	PRJNA1530935	5–15
L1.4	SL1.4	SL1.4	Laboratory-reared	First-instar larva	Midgut	4	SRR40720496	SAMN63217855	SRP737584	PRJNA1530935	5–15
L1.5	SL1.5	SL1.5	Laboratory-reared	First-instar larva	Midgut	5	SRR40720485	SAMN63217856	SRP737584	PRJNA1530935	5–15
L2.1	SL2.1	SL2.1	Laboratory-reared	Second-instar larva	Midgut	1	SRR40720474	SAMN63217857	SRP737584	PRJNA1530935	5–15
L2.2	SL2.2	SL2.2	Laboratory-reared	Second-instar larva	Midgut	2	SRR40720463	SAMN63217858	SRP737584	PRJNA1530935	5–15
L2.3	SL2.3	SL2.3	Laboratory-reared	Second-instar larva	Midgut	3	SRR40720452	SAMN63217859	SRP737584	PRJNA1530935	5–15
L2.4	SL2.4	SL2.4	Laboratory-reared	Second-instar larva	Midgut	4	SRR40720441	SAMN63217860	SRP737584	PRJNA1530935	5–15
L2.5	SL2.5	SL2.5	Laboratory-reared	Second-instar larva	Midgut	5	SRR40720439	SAMN63217861	SRP737584	PRJNA1530935	5–15
L3.1	SL3.1	SL3.1	Laboratory-reared	Third-instar larva	Midgut	1	SRR40720517	SAMN63217862	SRP737584	PRJNA1530935	5–15
L3.2	SL3.2	SL3.2	Laboratory-reared	Third-instar larva	Midgut	2	SRR40720516	SAMN63217863	SRP737584	PRJNA1530935	5–15
L3.3	SL3.3	SL3.3	Laboratory-reared	Third-instar larva	Midgut	3	SRR40720515	SAMN63217864	SRP737584	PRJNA1530935	5–15
L3.4	SL3.4	SL3.4	Laboratory-reared	Third-instar larva	Midgut	4	SRR40720514	SAMN63217865	SRP737584	PRJNA1530935	5–15
L3.5	SL3.5	SL3.5	Laboratory-reared	Third-instar larva	Midgut	5	SRR40720513	SAMN63217866	SRP737584	PRJNA1530935	5–15
L4.1	SL4.1	SL4.1	Laboratory-reared	Fourth-instar larva	Midgut	1	SRR40720512	SAMN63217867	SRP737584	PRJNA1530935	5–15
L4.2	SL4.2	SL4.2	Laboratory-reared	Fourth-instar larva	Midgut	2	SRR40720511	SAMN63217868	SRP737584	PRJNA1530935	5–15
L4.3	SL4.3	SL4.3	Laboratory-reared	Fourth-instar larva	Midgut	3	SRR40720510	SAMN63217869	SRP737584	PRJNA1530935	5–15
L4.4	SL4.4	SL4.4	Laboratory-reared	Fourth-instar larva	Midgut	4	SRR40720509	SAMN63217870	SRP737584	PRJNA1530935	5–15
L4.5	SL4.5	SL4.5	Laboratory-reared	Fourth-instar larva	Midgut	5	SRR40720508	SAMN63217871	SRP737584	PRJNA1530935	5–15
L5.1	SL5.1	SL5.1	Laboratory-reared	Fifth-instar larva	Midgut	1	SRR40720506	SAMN63217872	SRP737584	PRJNA1530935	5–15

L5.2	SL5.2	SL5.2	Laboratory-reared	Fifth-instar larva	Midgut	2	SRR40720505	SAMN63217873	SRP737584	PRJNA1530935	5–15
L5.3	SL5.3	SL5.3	Laboratory-reared	Fifth-instar larva	Midgut	3	SRR40720504	SAMN63217874	SRP737584	PRJNA1530935	5–15
L5.4	SL5.4	SL5.4	Laboratory-reared	Fifth-instar larva	Midgut	4	SRR40720503	SAMN63217875	SRP737584	PRJNA1530935	5–15
L5.5	SL5.5	SL5.5	Laboratory-reared	Fifth-instar larva	Midgut	5	SRR40720502	SAMN63217876	SRP737584	PRJNA1530935	5–15
Pu.1	P1	P1	Laboratory-reared	Pupa	Midgut	1	SRR40720501	SAMN63217877	SRP737584	PRJNA1530935	5–15
Pu.2	P2	P2	Laboratory-reared	Pupa	Midgut	2	SRR40720500	SAMN63217878	SRP737584	PRJNA1530935	5–15
Pu.3	P3	P3	Laboratory-reared	Pupa	Midgut	3	SRR40720499	SAMN63217879	SRP737584	PRJNA1530935	5–15
Pu.4	P4	P4	Laboratory-reared	Pupa	Midgut	4	SRR40720498	SAMN63217880	SRP737584	PRJNA1530935	5–15
Pu.5	P5	P5	Laboratory-reared	Pupa	Midgut	5	SRR40720497	SAMN63217881	SRP737584	PRJNA1530935	5–15
Ma.1	M1	M1	Laboratory-reared	Male adult	Midgut	1	SRR40720495	SAMN63217882	SRP737584	PRJNA1530935	5–15
Ma.2	M2	M2	Laboratory-reared	Male adult	Midgut	2	SRR40720494	SAMN63217883	SRP737584	PRJNA1530935	5–15
Ma.3	M3	M3	Laboratory-reared	Male adult	Midgut	3	SRR40720493	SAMN63217884	SRP737584	PRJNA1530935	5–15
Ma.4	M4	M4	Laboratory-reared	Male adult	Midgut	4	SRR40720492	SAMN63217885	SRP737584	PRJNA1530935	5–15
Ma.5	M5	M5	Laboratory-reared	Male adult	Midgut	5	SRR40720491	SAMN63217886	SRP737584	PRJNA1530935	5–15
Fe.1	F1	F1	Laboratory-reared	Female adult	Midgut	1	SRR40720490	SAMN63217887	SRP737584	PRJNA1530935	5–15
Fe.2	F2	F2	Laboratory-reared	Female adult	Midgut	2	SRR40720489	SAMN63217888	SRP737584	PRJNA1530935	5–15
Fe.3	F3	F3	Laboratory-reared	Female adult	Midgut	3	SRR40720488	SAMN63217889	SRP737584	PRJNA1530935	5–15
Fe.4	F4	F4	Laboratory-reared	Female adult	Midgut	4	SRR40720487	SAMN63217890	SRP737584	PRJNA1530935	5–15
Fe.5	F5	F5	Laboratory-reared	Female adult	Midgut	5	SRR40720486	SAMN63217891	SRP737584	PRJNA1530935	5–15
EL5.1	NE1	NE1	Laboratory-reared	Fifth-instar larva	Epidermis	1	SRR40720484	SAMN63217892	SRP737584	PRJNA1530935	5
EL5.2	NE2	NE2	Laboratory-reared	Fifth-instar larva	Epidermis	2	SRR40720483	SAMN63217893	SRP737584	PRJNA1530935	5
EL5.3	NE3	NE3	Laboratory-reared	Fifth-instar larva	Epidermis	3	SRR40720482	SAMN63217894	SRP737584	PRJNA1530935	5
EL5.4	NE4	NE4	Laboratory-reared	Fifth-instar larva	Epidermis	4	SRR40720481	SAMN63217895	SRP737584	PRJNA1530935	5

FL5.1	NF1	NF1	Laboratory-reared	Fifth-instar larva	Frass	1	SRR40720480	SAMN63217896	SRP737584	PRJNA1530935	10
FL5.2	NF2	NF2	Laboratory-reared	Fifth-instar larva	Frass	2	SRR40720479	SAMN63217897	SRP737584	PRJNA1530935	10
FL5.3	NF3	NF3	Laboratory-reared	Fifth-instar larva	Frass	3	SRR40720478	SAMN63217898	SRP737584	PRJNA1530935	10
FL5.4	NF4	NF4	Laboratory-reared	Fifth-instar larva	Frass	4	SRR40720477	SAMN63217899	SRP737584	PRJNA1530935	10
ML5.1	NM1	NM1	Laboratory-reared	Fifth-instar larva	Midgut	1	SRR40720476	SAMN63217900	SRP737584	PRJNA1530935	5
ML5.2	NM2	NM2	Laboratory-reared	Fifth-instar larva	Midgut	2	SRR40720475	SAMN63217901	SRP737584	PRJNA1530935	5
ML5.3	NM3	NM3	Laboratory-reared	Fifth-instar larva	Midgut	3	SRR40720473	SAMN63217902	SRP737584	PRJNA1530935	5
ML5.4	NM4	NM4	Laboratory-reared	Fifth-instar larva	Midgut	4	SRR40720472	SAMN63217903	SRP737584	PRJNA1530935	5
OL5.1	NO1	NO1	Laboratory-reared	Fifth-instar larva	Other internal tissues	1	SRR40720471	SAMN63217904	SRP737584	PRJNA1530935	5
OL5.2	NO2	NO2	Laboratory-reared	Fifth-instar larva	Other internal tissues	2	SRR40720470	SAMN63217905	SRP737584	PRJNA1530935	5
OL5.3	NO3	NO3	Laboratory-reared	Fifth-instar larva	Other internal tissues	3	SRR40720469	SAMN63217906	SRP737584	PRJNA1530935	5
OL5.4	NO4	NO4	Laboratory-reared	Fifth-instar larva	Other internal tissues	4	SRR40720468	SAMN63217907	SRP737584	PRJNA1530935	5
WL1.1	WL1.1	WL2.1	Field-collected	First-instar larva	Midgut	1	SRR40720467	SAMN63217908	SRP737584	PRJNA1530935	5–15
WL1.2	WL1.2	WL2.2	Field-collected	First-instar larva	Midgut	2	SRR40720466	SAMN63217909	SRP737584	PRJNA1530935	5–15
WL1.3	WL1.3	WL2.3	Field-collected	First-instar larva	Midgut	3	SRR40720465	SAMN63217910	SRP737584	PRJNA1530935	5–15
WL1.4	WL1.4	WL2.4	Field-collected	First-instar larva	Midgut	4	SRR40720464	SAMN63217911	SRP737584	PRJNA1530935	5–15
WL1.5	WL1.5	WL2.5	Field-collected	First-instar larva	Midgut	5	SRR40720462	SAMN63217912	SRP737584	PRJNA1530935	5–15
WL2.1	WL2.1	WL3.1	Field-collected	Second-instar larva	Midgut	1	SRR40720461	SAMN63217913	SRP737584	PRJNA1530935	5–15
WL2.2	WL2.2	WL3.2	Field-collected	Second-instar larva	Midgut	2	SRR40720460	SAMN63217914	SRP737584	PRJNA1530935	5–15
WL2.3	WL2.3	WL3.3	Field-collected	Second-instar larva	Midgut	3	SRR40720459	SAMN63217915	SRP737584	PRJNA1530935	5–15
WL2.4	WL2.4	WL3.4	Field-collected	Second-instar larva	Midgut	4	SRR40720458	SAMN63217916	SRP737584	PRJNA1530935	5–15
WL2.5	WL2.5	WL3.5	Field-collected	Second-instar larva	Midgut	5	SRR40720457	SAMN63217917	SRP737584	PRJNA1530935	5–15
WL3.1	WL3.1	WL4.1	Field-collected	Third-instar larva	Midgut	1	SRR40720456	SAMN63217918	SRP737584	PRJNA1530935	5–15

WL3.2	WL3.2	WL4.2	Field-collected	Third-instar larva	Midgut	2	SRR40720455	SAMN63217919	SRP737584	PRJNA1530935	5–15
WL3.3	WL3.3	WL4.3	Field-collected	Third-instar larva	Midgut	3	SRR40720454	SAMN63217920	SRP737584	PRJNA1530935	5–15
WL3.4	WL3.4	WL4.4	Field-collected	Third-instar larva	Midgut	4	SRR40720453	SAMN63217921	SRP737584	PRJNA1530935	5–15
WL3.5	WL3.5	WL4.5	Field-collected	Third-instar larva	Midgut	5	SRR40720451	SAMN63217922	SRP737584	PRJNA1530935	5–15
WL4.1	WL4.1	WL5.1	Field-collected	Fourth-instar larva	Midgut	1	SRR40720450	SAMN63217923	SRP737584	PRJNA1530935	5–15
WL4.2	WL4.2	WL5.2	Field-collected	Fourth-instar larva	Midgut	2	SRR40720449	SAMN63217924	SRP737584	PRJNA1530935	5–15
WL4.3	WL4.3	WL5.3	Field-collected	Fourth-instar larva	Midgut	3	SRR40720448	SAMN63217925	SRP737584	PRJNA1530935	5–15
WL4.4	WL4.4	WL5.4	Field-collected	Fourth-instar larva	Midgut	4	SRR40720447	SAMN63217926	SRP737584	PRJNA1530935	5–15
WL4.5	WL4.5	WL5.5	Field-collected	Fourth-instar larva	Midgut	5	SRR40720446	SAMN63217927	SRP737584	PRJNA1530935	5–15
WL5.1	WL5.1	WL6.1	Field-collected	Fifth-instar larva	Midgut	1	SRR40720445	SAMN63217928	SRP737584	PRJNA1530935	5–15
WL5.2	WL5.2	WL6.2	Field-collected	Fifth-instar larva	Midgut	2	SRR40720444	SAMN63217929	SRP737584	PRJNA1530935	5–15
WL5.3	WL5.3	WL6.3	Field-collected	Fifth-instar larva	Midgut	3	SRR40720443	SAMN63217930	SRP737584	PRJNA1530935	5–15
WL5.4	WL5.4	WL6.4	Field-collected	Fifth-instar larva	Midgut	4	SRR40720442	SAMN63217931	SRP737584	PRJNA1530935	5–15
WL5.5	WL5.5	WL6.5	Field-collected	Fifth-instar larva	Midgut	5	SRR40720440	SAMN63217932	SRP737584	PRJNA1530935	5–15

Notes

Analysis unit Each sample is one pooled biological replicate and one unit of analysis. Laboratory developmental-stage/adult-sex groups and field instars each have five pooled biological replicates. Each tissue/frass type has four pooled biological replicates. Individuals within a pool are not counted as separate replicates.

Pool size For laboratory developmental-stage/adult-sex and field-instar samples, each replicate contains midguts from 5–15 individuals. Pool size varies with body size to obtain sufficient material and DNA. For the fifth-instar tissue comparison, each midgut, epidermis or other-internal-tissue replicate pools the corresponding material from five larvae. Each frass replicate pools frass from ten larvae. Different sample types originate from different insects. The range 5–15 reports the confirmed pooling range, not an exact individual count for each sample.

Female lineages Five independent female lineages were established for the laboratory developmental-stage/adult-sex comparison. Each group contains one pooled sample from each lineage. Individuals within a pool originate from the same female lineage, and the five pools within a group originate from five different female lineages.

Identifiers L1–L5: first- to fifth-instar laboratory larvae. Pu: pupae. Ma: male adults. Fe: female adults. WL1–WL5: first- to fifth-instar field larvae. ML/ML5: midgut. EL/EL5: epidermis. OL/OL5: other internal tissues. FL/FL5: frass. Company WL2–WL6 correspond to manuscript WL1–WL5. Submitted sample names retain the original deposition metadata.

Field collection Field larvae were collected from Chenopodium album in May 2023 in Zhengxiangbai Banner, Xilin Gol League, Inner Mongolia, China (42.210876° N, 114.891301° E). Larvae were transported alive for indoor processing. Dissected midguts were immediately stored at −80 °C.

Rearing and tissues Laboratory larvae were fed fresh Chenopodium album leaves in sterilized containers. Midgut samples contain the midgut and its contents. Other internal tissues are the internal tissues remaining after midgut and epidermis removal. Epidermis is the body covering remaining after removal of internal tissues and was rinsed with PBS. Frass was collected after 6 h of normal feeding in sterilized containers.

Data deposition All 81 pooled samples are linked to individual SRA run and BioSample accessions under BioProject PRJNA1530935 and SRA study SRP737584. The deposited data are currently held for release and will be made publicly available upon publication. BioProject: https://www.ncbi.nlm.nih.gov/bioproject/PRJNA1530935

Functional prediction Functional heatmaps were generated with the PICRUSt v1.1.4 module in NovoMagic, using Greengenes v13.5, KO annotations and KEGG level 2. Comparisons are descriptive. NSTI values were not available in the accessible exported files for this cloud task.

Source records Sample mapping and individual accessions are retained from the supplied SRA metadata. Sampling and pooling descriptions follow the author-confirmed experimental design.

Table S3. Cyanobacteria-assigned ASVs excluded by taxonomic filtering

Dataset	ASV ID	Taxonomic annotation	Decision	Exclusion rule	Sequences removed in manuscript samples
lab	ASV2	k__Bacteria;p__Cyanobacteria;c__Cyanobacteriia;o__Chloroplast;f__unidentified_Chloroplast;g__unidentified_Chloroplast	excluded	phylum_Cyanobacteria	231,847
lab	ASV39	k__Bacteria;p__Cyanobacteria;c__Cyanobacteriia;o__Chloroplast;f__unidentified_Chloroplast;g__unidentified_Chloroplast	excluded	phylum_Cyanobacteria	10,811
lab	ASV159	k__Bacteria;p__Cyanobacteria;c__Cyanobacteriia;o__Chloroplast;f__unidentified_Chloroplast;g__unidentified_Chloroplast	excluded	phylum_Cyanobacteria	1,042
lab	ASV177	k__Bacteria;p__Cyanobacteria;c__Cyanobacteriia;o__Chloroplast;f__unidentified_Chloroplast;g__unidentified_Chloroplast	excluded	phylum_Cyanobacteria	883
lab	ASV251	k__Bacteria;p__Cyanobacteria;c__Cyanobacteriia;o__Chloroplast;f__unidentified_Chloroplast;g__unidentified_Chloroplast	excluded	phylum_Cyanobacteria	587
lab	ASV266	k__Bacteria;p__Cyanobacteria;c__Cyanobacteriia;o__Chloroplast;f__unidentified_Chloroplast;g__unidentified_Chloroplast	excluded	phylum_Cyanobacteria	549
lab	ASV314	k__Bacteria;p__Cyanobacteria;c__Cyanobacteriia;o__Chloroplast;f__unidentified_Chloroplast;g__unidentified_Chloroplast	excluded	phylum_Cyanobacteria	449
lab	ASV351	k__Bacteria;p__Cyanobacteria;c__Cyanobacteriia;o__Chloroplast;f__unidentified_Chloroplast;g__unidentified_Chloroplast	excluded	phylum_Cyanobacteria	395
lab	ASV357	k__Bacteria;p__Cyanobacteria;c__Cyanobacteriia;o__Chloroplast;f__unidentified_Chloroplast;g__unidentified_Chloroplast	excluded	phylum_Cyanobacteria	389
lab	ASV409	k__Bacteria;p__Cyanobacteria;c__Cyanobacteriia;o__Chloroplast;f__unidentified_Chloroplast;g__unidentified_Chloroplast	excluded	phylum_Cyanobacteria	347
lab	ASV424	k__Bacteria;p__Cyanobacteria;c__Cyanobacteriia;o__Chloroplast;f__unidentified_Chloroplast;g__unidentified_Chloroplast;s__Planoglabratella_opercul aris	excluded	phylum_Cyanobacteria	335
lab	ASV505	k__Bacteria;p__Cyanobacteria;c__Cyanobacteriia;o__Chloroplast;f__unidentified_Chloroplast;g__unidentified_Chloroplast	excluded	phylum_Cyanobacteria	289
lab	ASV721	k__Bacteria;p__Cyanobacteria;c__Cyanobacteriia;o__Chloroplast;f__unidentified_Chloroplast;g__unidentified_Chloroplast	excluded	phylum_Cyanobacteria	201
lab	ASV727	k__Bacteria;p__Cyanobacteria;c__Cyanobacteriia;o__Chloroplast;f__unidentified_Chloroplast;g__unidentified_Chloroplast	excluded	phylum_Cyanobacteria	201
lab	ASV761	k__Bacteria;p__Cyanobacteria;c__Cyanobacteriia;o__Chloroplast;f__unidentified_Chloroplast;g__unidentified_Chloroplast	excluded	phylum_Cyanobacteria	194
lab	ASV763	k__Bacteria;p__Cyanobacteria;c__Cyanobacteriia;o__Chloroplast;f__unidentified_Chloroplast;g__unidentified_Chloroplast	excluded	phylum_Cyanobacteria	193
lab	ASV820	k__Bacteria;p__Cyanobacteria;c__Cyanobacteriia;o__Chloroplast;f__unidentified_Chloroplast;g__unidentified_Chloroplast	excluded	phylum_Cyanobacteria	178
lab	ASV840	k__Bacteria;p__Cyanobacteria;c__Cyanobacteriia;o__Chloroplast;f__unidentified_Chloroplast;g__unidentified_Chloroplast	excluded	phylum_Cyanobacteria	173
lab	ASV846	k__Bacteria;p__Cyanobacteria;c__Cyanobacteriia;o__Chloroplast;f__unidentified_Chloroplast;g__unidentified_Chloroplast	excluded	phylum_Cyanobacteria	171
lab	ASV871	k__Bacteria;p__Cyanobacteria;c__Cyanobacteriia;o__Chloroplast;f__unidentified_Chloroplast;g__unidentified_Chloroplast	excluded	phylum_Cyanobacteria	167
lab	ASV885	k__Bacteria;p__Cyanobacteria;c__Cyanobacteriia;o__Chloroplast;f__unidentified_Chloroplast;g__unidentified_Chloroplast	excluded	phylum_Cyanobacteria	165
lab	ASV948	k__Bacteria;p__Cyanobacteria;c__Cyanobacteriia;o__Chloroplast;f__unidentified_Chloroplast;g__unidentified_Chloroplast	excluded	phylum_Cyanobacteria	153
lab	ASV967	k__Bacteria;p__Cyanobacteria;c__Cyanobacteriia;o__Chloroplast;f__unidentified_Chloroplast;g__unidentified_Chloroplast	excluded	phylum_Cyanobacteria	149
lab	ASV1056	k__Bacteria;p__Cyanobacteria;c__Cyanobacteriia;o__Chloroplast;f__unidentified_Chloroplast;g__unidentified_Chloroplast	excluded	phylum_Cyanobacteria	137
lab	ASV1067	k__Bacteria;p__Cyanobacteria;c__Cyanobacteriia;o__Chloroplast;f__unidentified_Chloroplast;g__unidentified_Chloroplast	excluded	phylum_Cyanobacteria	136
lab	ASV1075	k__Bacteria;p__Cyanobacteria;c__Cyanobacteriia;o__Chloroplast;f__unidentified_Chloroplast;g__unidentified_Chloroplast	excluded	phylum_Cyanobacteria	134
lab	ASV1111	k__Bacteria;p__Cyanobacteria;c__Cyanobacteriia;o__Chloroplast;f__unidentified_Chloroplast;g__unidentified_Chloroplast	excluded	phylum_Cyanobacteria	131
lab	ASV1165	k__Bacteria;p__Cyanobacteria;c__Vampirivibrionia;o__Obscuribacterales;f__Obscuribacteraceae;g__;s__	excluded	phylum_Cyanobacteria	124
lab	ASV1168	k__Bacteria;p__Cyanobacteria;c__Cyanobacteriia;o__Chloroplast;f__unidentified_Chloroplast;g__unidentified_Chloroplast	excluded	phylum_Cyanobacteria	123
lab	ASV1179	k__Bacteria;p__Cyanobacteria;c__Cyanobacteriia;o__Chloroplast;f__unidentified_Chloroplast;g__unidentified_Chloroplast	excluded	phylum_Cyanobacteria	122
lab	ASV1190	k__Bacteria;p__Cyanobacteria;c__Cyanobacteriia;o__Chloroplast;f__unidentified_Chloroplast;g__unidentified_Chloroplast	excluded	phylum_Cyanobacteria	121
lab	ASV1285	k__Bacteria;p__Cyanobacteria;c__Cyanobacteriia;o__Chloroplast;f__unidentified_Chloroplast;g__unidentified_Chloroplast	excluded	phylum_Cyanobacteria	111
lab	ASV1348	k__Bacteria;p__Cyanobacteria;c__Cyanobacteriia;o__Chloroplast;f__unidentified_Chloroplast;g__unidentified_Chloroplast;s__Guillardia_theta	excluded	phylum_Cyanobacteria	105
lab	ASV1381	k__Bacteria;p__Cyanobacteria;c__Cyanobacteriia;o__Chloroplast;f__unidentified_Chloroplast;g__unidentified_Chloroplast	excluded	phylum_Cyanobacteria	102
lab	ASV1410	k__Bacteria;p__Cyanobacteria;c__Cyanobacteriia;o__Chloroplast;f__unidentified_Chloroplast;g__unidentified_Chloroplast	excluded	phylum_Cyanobacteria	100
lab	ASV1420	k__Bacteria;p__Cyanobacteria;c__Vampirivibrionia;o__Obscuribacterales;f__Obscuribacteraceae;g__Candidatus_Obscuribacter;s__	excluded	phylum_Cyanobacteria	100
lab	ASV1424	k__Bacteria;p__Cyanobacteria;c__Cyanobacteriia;o__Chloroplast;f__unidentified_Chloroplast;g__unidentified_Chloroplast	excluded	phylum_Cyanobacteria	99

lab	ASV1434	k__Bacteria;p__Cyanobacteria;c__Cyanobacteriia;o__Chloroplast;f__unidentified_Chloroplast;g__unidentified_Chloroplast	excluded	phylum_Cyanobacteria	98
lab	ASV1449	k__Bacteria;p__Cyanobacteria;c__Cyanobacteriia;o__Chloroplast;f__unidentified_Chloroplast;g__unidentified_Chloroplast	excluded	phylum_Cyanobacteria	98
lab	ASV1450	k__Bacteria;p__Cyanobacteria;c__Cyanobacteriia;o__Chloroplast;f__unidentified_Chloroplast;g__unidentified_Chloroplast	excluded	phylum_Cyanobacteria	97
lab	ASV1493	k__Bacteria;p__Cyanobacteria;c__Cyanobacteriia;o__Chloroplast;f__unidentified_Chloroplast;g__unidentified_Chloroplast	excluded	phylum_Cyanobacteria	94
lab	ASV1568	k__Bacteria;p__Cyanobacteria;c__Cyanobacteriia;o__Chloroplast;f__unidentified_Chloroplast;g__unidentified_Chloroplast	excluded	phylum_Cyanobacteria	89
lab	ASV1600	k__Bacteria;p__Cyanobacteria;c__Cyanobacteriia;o__Chloroplast;f__unidentified_Chloroplast;g__unidentified_Chloroplast	excluded	phylum_Cyanobacteria	87
lab	ASV1735	k__Bacteria;p__Cyanobacteria;c__Cyanobacteriia;o__Chloroplast;f__unidentified_Chloroplast;g__unidentified_Chloroplast	excluded	phylum_Cyanobacteria	79
lab	ASV1774	k__Bacteria;p__Cyanobacteria;c__Cyanobacteriia;o__Chloroplast;f__unidentified_Chloroplast;g__unidentified_Chloroplast	excluded	phylum_Cyanobacteria	77
lab	ASV1869	k__Bacteria;p__Cyanobacteria;c__Cyanobacteriia;o__Chloroplast;f__unidentified_Chloroplast;g__unidentified_Chloroplast	excluded	phylum_Cyanobacteria	72
lab	ASV1916	k__Bacteria;p__Cyanobacteria;c__Cyanobacteriia;o__Chloroplast;f__unidentified_Chloroplast;g__unidentified_Chloroplast	excluded	phylum_Cyanobacteria	70
lab	ASV1953	k__Bacteria;p__Cyanobacteria;c__Cyanobacteriia;o__Chloroplast;f__unidentified_Chloroplast;g__unidentified_Chloroplast	excluded	phylum_Cyanobacteria	68
lab	ASV1992	k__Bacteria;p__Cyanobacteria;c__Cyanobacteriia;o__Chloroplast;f__unidentified_Chloroplast;g__unidentified_Chloroplast	excluded	phylum_Cyanobacteria	66
lab	ASV1995	k__Bacteria;p__Cyanobacteria;c__Cyanobacteriia;o__Chloroplast;f__unidentified_Chloroplast;g__unidentified_Chloroplast	excluded	phylum_Cyanobacteria	66
lab	ASV2006	k__Bacteria;p__Cyanobacteria;c__Cyanobacteriia;o__Chloroplast;f__unidentified_Chloroplast;g__unidentified_Chloroplast	excluded	phylum_Cyanobacteria	66
lab	ASV2096	k__Bacteria;p__Cyanobacteria;c__Cyanobacteriia;o__Chloroplast;f__unidentified_Chloroplast;g__unidentified_Chloroplast	excluded	phylum_Cyanobacteria	62
lab	ASV2097	k__Bacteria;p__Cyanobacteria;c__Cyanobacteriia;o__Chloroplast;f__unidentified_Chloroplast;g__unidentified_Chloroplast	excluded	phylum_Cyanobacteria	62
lab	ASV2228	k__Bacteria;p__Cyanobacteria;c__Cyanobacteriia;o__Chloroplast;f__unidentified_Chloroplast;g__unidentified_Chloroplast	excluded	phylum_Cyanobacteria	57
lab	ASV2236	k__Bacteria;p__Cyanobacteria;c__Cyanobacteriia;o__Cyanobacteriales;f__Chroococcidiopsaceae;g__Gloeocapsa_PCC-7428;s__	excluded	phylum_Cyanobacteria	57
lab	ASV2373	k__Bacteria;p__Cyanobacteria;c__Cyanobacteriia;o__Chloroplast;f__unidentified_Chloroplast;g__unidentified_Chloroplast	excluded	phylum_Cyanobacteria	52
lab	ASV2405	k__Bacteria;p__Cyanobacteria;c__Vampirivibrionia;o__Obscuribacterales;f__Obscuribacteraceae;g__Candidatus_Obscuribacter;s__	excluded	phylum_Cyanobacteria	51
lab	ASV2635	k__Bacteria;p__Cyanobacteria;c__Cyanobacteriia;o__Chloroplast;f__unidentified_Chloroplast;g__unidentified_Chloroplast	excluded	phylum_Cyanobacteria	45
lab	ASV2729	k__Bacteria;p__Cyanobacteria;c__Cyanobacteriia;o__Chloroplast;f__unidentified_Chloroplast;g__unidentified_Chloroplast	excluded	phylum_Cyanobacteria	43
lab	ASV2794	k__Bacteria;p__Cyanobacteria;c__Cyanobacteriia;o__Chloroplast;f__unidentified_Chloroplast;g__unidentified_Chloroplast	excluded	phylum_Cyanobacteria	41
lab	ASV2795	k__Bacteria;p__Cyanobacteria;c__Cyanobacteriia;o__Chloroplast;f__unidentified_Chloroplast;g__unidentified_Chloroplast	excluded	phylum_Cyanobacteria	41
lab	ASV2864	k__Bacteria;p__Cyanobacteria;c__Cyanobacteriia;o__Chloroplast;f__unidentified_Chloroplast;g__unidentified_Chloroplast	excluded	phylum_Cyanobacteria	39
lab	ASV3041	k__Bacteria;p__Cyanobacteria;c__Cyanobacteriia;o__Chloroplast;f__unidentified_Chloroplast;g__unidentified_Chloroplast	excluded	phylum_Cyanobacteria	35
lab	ASV3252	k__Bacteria;p__Cyanobacteria;c__Cyanobacteriia;o__Chloroplast;f__unidentified_Chloroplast;g__unidentified_Chloroplast;s__Nicotiana_tomentosiformis	excluded	phylum_Cyanobacteria	31
lab	ASV3304	k__Bacteria;p__Cyanobacteria;c__Cyanobacteriia;o__Chloroplast;f__unidentified_Chloroplast;g__unidentified_Chloroplast	excluded	phylum_Cyanobacteria	30
lab	ASV3306	k__Bacteria;p__Cyanobacteria;c__Cyanobacteriia;o__Chloroplast;f__unidentified_Chloroplast;g__unidentified_Chloroplast	excluded	phylum_Cyanobacteria	30
lab	ASV3360	k__Bacteria;p__Cyanobacteria;c__Cyanobacteriia;o__Chloroplast;f__unidentified_Chloroplast;g__unidentified_Chloroplast	excluded	phylum_Cyanobacteria	29
lab	ASV3367	k__Bacteria;p__Cyanobacteria;c__Cyanobacteriia;o__Chloroplast;f__unidentified_Chloroplast;g__unidentified_Chloroplast	excluded	phylum_Cyanobacteria	29
lab	ASV3416	k__Bacteria;p__Cyanobacteria;c__Cyanobacteriia;o__Chloroplast;f__unidentified_Chloroplast;g__unidentified_Chloroplast	excluded	phylum_Cyanobacteria	28
lab	ASV3518	k__Bacteria;p__Cyanobacteria;c__Cyanobacteriia;o__Chloroplast;f__unidentified_Chloroplast;g__unidentified_Chloroplast	excluded	phylum_Cyanobacteria	26
lab	ASV3699	k__Bacteria;p__Cyanobacteria;c__Cyanobacteriia;o__Chloroplast;f__unidentified_Chloroplast;g__unidentified_Chloroplast	excluded	phylum_Cyanobacteria	23
lab	ASV3875	k__Bacteria;p__Cyanobacteria;c__Cyanobacteriia;o__Chloroplast;f__unidentified_Chloroplast;g__unidentified_Chloroplast	excluded	phylum_Cyanobacteria	21
lab	ASV4026	k__Bacteria;p__Cyanobacteria;c__Cyanobacteriia;o__Chloroplast;f__unidentified_Chloroplast;g__unidentified_Chloroplast	excluded	phylum_Cyanobacteria	19
lab	ASV4297	k__Bacteria;p__Cyanobacteria;c__Cyanobacteriia;o__Cyanobacteriales;f__Nostocaceae;g__Synechococcus_PCC-7902;s__Synechococcus_sp_PCC_7902	excluded	phylum_Cyanobacteria	16

lab	ASV4326	k__Bacteria;p__Cyanobacteria;c__Vampirivibrionia;o__Gastranaerophilales;f__unidentified_Gastranaerophilales;g__unidentified_Gastranaerophilales;s__Candidatus_Gastranaerophilales_bacterium_Zag_111	excluded	phylum_Cyanobacteria	15
lab	ASV4519	k__Bacteria;p__Cyanobacteria;c__Cyanobacteriia;o__Cyanobacteriales	excluded	phylum_Cyanobacteria	14
lab	ASV4660	k__Bacteria;p__Cyanobacteria;c__Cyanobacteriia;o__Cyanobacteriales;f__Xenococcaceae;g__Pleurocapsa_PCC-7319	excluded	phylum_Cyanobacteria	12
lab	ASV4708	k__Bacteria;p__Cyanobacteria;c__Cyanobacteriia;o__Chloroplast;f__unidentified_Chloroplast;g__unidentified_Chloroplast	excluded	phylum_Cyanobacteria	12
lab	ASV4761	k__Bacteria;p__Cyanobacteria;c__Sericytochromatia;o__;f__;g__;s__	excluded	phylum_Cyanobacteria	12
lab	ASV4819	k__Bacteria;p__Cyanobacteria;c__Cyanobacteriia;o__Chloroplast;f__unidentified_Chloroplast;g__unidentified_Chloroplast	excluded	phylum_Cyanobacteria	11
lab	ASV4961	k__Bacteria;p__Cyanobacteria;c__Vampirivibrionia;o__Obscuribacterales;f__Obscuribacteraceae;g__Candidatus_Obscuribacter;s__	excluded	phylum_Cyanobacteria	10
lab	ASV5024	k__Bacteria;p__Cyanobacteria;c__Cyanobacteriia;o__Chloroplast;f__unidentified_Chloroplast;g__unidentified_Chloroplast	excluded	phylum_Cyanobacteria	10
lab	ASV5074	k__Bacteria;p__Cyanobacteria;c__Cyanobacteriia;o__Chloroplast;f__unidentified_Chloroplast;g__unidentified_Chloroplast	excluded	phylum_Cyanobacteria	9
lab	ASV5976	k__Bacteria;p__Cyanobacteria;c__Cyanobacteriia;o__Leptolyngbyales;f__Leptolyngbyaceae;g__;s__	excluded	phylum_Cyanobacteria	5
lab	ASV6799	k__Bacteria;p__Cyanobacteria;c__Cyanobacteriia;o__Cyanobacteriales;f__Xenococcaceae;g__Pleurocapsa_PCC-7319	excluded	phylum_Cyanobacteria	3
tissue	ASV2	k__Bacteria;p__Cyanobacteria;c__Cyanobacteriia;o__Chloroplast;f__unidentified_Chloroplast;g__unidentified_Chloroplast	excluded	phylum_Cyanobacteria	121,672
tissue	ASV78	k__Bacteria;p__Cyanobacteria;c__Cyanobacteriia;o__Chloroplast;f__unidentified_Chloroplast;g__unidentified_Chloroplast	excluded	phylum_Cyanobacteria	1,729
tissue	ASV211	k__Bacteria;p__Cyanobacteria;c__Cyanobacteriia;o__Chloroplast;f__unidentified_Chloroplast;g__unidentified_Chloroplast	excluded	phylum_Cyanobacteria	388
tissue	ASV215	k__Bacteria;p__Cyanobacteria;c__Cyanobacteriia;o__Chloroplast;f__unidentified_Chloroplast;g__unidentified_Chloroplast	excluded	phylum_Cyanobacteria	382
tissue	ASV273	k__Bacteria;p__Cyanobacteria;c__Cyanobacteriia;o__Chloroplast;f__unidentified_Chloroplast;g__unidentified_Chloroplast	excluded	phylum_Cyanobacteria	272
tissue	ASV293	k__Bacteria;p__Cyanobacteria;c__Cyanobacteriia;o__Chloroplast;f__unidentified_Chloroplast;g__unidentified_Chloroplast	excluded	phylum_Cyanobacteria	244
tissue	ASV313	k__Bacteria;p__Cyanobacteria;c__Cyanobacteriia;o__Chloroplast;f__unidentified_Chloroplast;g__unidentified_Chloroplast	excluded	phylum_Cyanobacteria	231
tissue	ASV413	k__Bacteria;p__Cyanobacteria;c__Cyanobacteriia;o__Chloroplast;f__unidentified_Chloroplast;g__unidentified_Chloroplast	excluded	phylum_Cyanobacteria	163
tissue	ASV601	k__Bacteria;p__Cyanobacteria;c__Cyanobacteriia;o__Chloroplast;f__unidentified_Chloroplast;g__unidentified_Chloroplast	excluded	phylum_Cyanobacteria	0
tissue	ASV628	k__Bacteria;p__Cyanobacteria;c__Cyanobacteriia;o__Chloroplast;f__unidentified_Chloroplast;g__unidentified_Chloroplast	excluded	phylum_Cyanobacteria	104
tissue	ASV777	k__Bacteria;p__Cyanobacteria;c__Cyanobacteriia;o__Chloroplast;f__unidentified_Chloroplast;g__unidentified_Chloroplast	excluded	phylum_Cyanobacteria	82
tissue	ASV794	k__Bacteria;p__Cyanobacteria;c__Sericytochromatia;o__unidentified_Sericytochromatia;f__unidentified_Sericytochromatia;g__unidentified_Sericytochromatia;s__Hyaloperonospora_arabidopsidis	excluded	phylum_Cyanobacteria	80
tissue	ASV883	k__Bacteria;p__Cyanobacteria;c__Cyanobacteriia;o__Chloroplast;f__unidentified_Chloroplast;g__unidentified_Chloroplast	excluded	phylum_Cyanobacteria	73
tissue	ASV1065	k__Bacteria;p__Cyanobacteria;c__Cyanobacteriia;o__Chloroplast;f__unidentified_Chloroplast;g__unidentified_Chloroplast	excluded	phylum_Cyanobacteria	58
tissue	ASV1083	k__Bacteria;p__Cyanobacteria;c__Cyanobacteriia;o__Chloroplast;f__unidentified_Chloroplast;g__unidentified_Chloroplast	excluded	phylum_Cyanobacteria	57
tissue	ASV1090	k__Bacteria;p__Cyanobacteria;c__Cyanobacteriia;o__Chloroplast;f__unidentified_Chloroplast;g__unidentified_Chloroplast;s__Poterioochromonas_sp_DS	excluded	phylum_Cyanobacteria	57
tissue	ASV1127	k__Bacteria;p__Cyanobacteria;c__Cyanobacteriia;o__Chloroplast;f__unidentified_Chloroplast;g__unidentified_Chloroplast	excluded	phylum_Cyanobacteria	55
tissue	ASV1182	k__Bacteria;p__Cyanobacteria;c__Vampirivibrionia;o__Obscuribacterales;f__Obscuribacteraceae;g__;s__	excluded	phylum_Cyanobacteria	52
tissue	ASV1279	k__Bacteria;p__Cyanobacteria;c__Cyanobacteriia;o__Chloroplast;f__unidentified_Chloroplast;g__unidentified_Chloroplast	excluded	phylum_Cyanobacteria	47
tissue	ASV1484	k__Bacteria;p__Cyanobacteria;c__Cyanobacteriia;o__Chloroplast;f__unidentified_Chloroplast;g__unidentified_Chloroplast	excluded	phylum_Cyanobacteria	39
tissue	ASV1520	k__Bacteria;p__Cyanobacteria;c__Cyanobacteriia;o__Chloroplast;f__unidentified_Chloroplast;g__unidentified_Chloroplast	excluded	phylum_Cyanobacteria	38
tissue	ASV1544	k__Bacteria;p__Cyanobacteria;c__Cyanobacteriia;o__Chloroplast;f__unidentified_Chloroplast;g__unidentified_Chloroplast	excluded	phylum_Cyanobacteria	37
tissue	ASV1554	k__Bacteria;p__Cyanobacteria;c__Cyanobacteriia;o__Cyanobacteriales;f__Phormidiaceae;g__Tychonema_CCAP_1459-11B	excluded	phylum_Cyanobacteria	0
tissue	ASV1583	k__Bacteria;p__Cyanobacteria;c__Cyanobacteriia;o__Chloroplast;f__unidentified_Chloroplast;g__unidentified_Chloroplast	excluded	phylum_Cyanobacteria	36
tissue	ASV1596	k__Bacteria;p__Cyanobacteria;c__Vampirivibrionia;o__Vampirovibrionales;f__Vampirovibrionaceae;g__;s__	excluded	phylum_Cyanobacteria	36
tissue	ASV1628	k__Bacteria;p__Cyanobacteria;c__Cyanobacteriia;o__Chloroplast;f__unidentified_Chloroplast;g__unidentified_Chloroplast	excluded	phylum_Cyanobacteria	35

tissue	ASV1711	k__Bacteria;p__Cyanobacteria;c__Cyanobacteriia;o__Cyanobacteriales;f__Phormidiaceae;g__Tychonema_CCAP_1459-11B	excluded	phylum_Cyanobacteria	32
tissue	ASV1748	k__Bacteria;p__Cyanobacteria;c__Vampirivibrionia;o__Vampirovibrionales;f__Vampirovibrionaceae;g__Vampirovibrio;s__	excluded	phylum_Cyanobacteria	31
tissue	ASV1832	k__Bacteria;p__Cyanobacteria;c__Cyanobacteriia;o__Cyanobacteriales;f__;g__;s__	excluded	phylum_Cyanobacteria	0
tissue	ASV1881	k__Bacteria;p__Cyanobacteria;c__Cyanobacteriia;o__Chloroplast;f__unidentified_Chloroplast;g__unidentified_Chloroplast	excluded	phylum_Cyanobacteria	27
tissue	ASV1972	k__Bacteria;p__Cyanobacteria;c__Cyanobacteriia;o__Chloroplast;f__unidentified_Chloroplast;g__unidentified_Chloroplast	excluded	phylum_Cyanobacteria	0
tissue	ASV2119	k__Bacteria;p__Cyanobacteria;c__Cyanobacteriia;o__Chloroplast;f__unidentified_Chloroplast;g__unidentified_Chloroplast	excluded	phylum_Cyanobacteria	22
tissue	ASV2135	k__Bacteria;p__Cyanobacteria;c__Cyanobacteriia;o__Chloroplast;f__unidentified_Chloroplast;g__unidentified_Chloroplast	excluded	phylum_Cyanobacteria	22
tissue	ASV2180	k__Bacteria;p__Cyanobacteria;c__Cyanobacteriia;o__Chloroplast;f__unidentified_Chloroplast;g__unidentified_Chloroplast	excluded	phylum_Cyanobacteria	21
tissue	ASV2235	k__Bacteria;p__Cyanobacteria;c__Cyanobacteriia;o__Chloroplast;f__unidentified_Chloroplast;g__unidentified_Chloroplast	excluded	phylum_Cyanobacteria	20
tissue	ASV2401	k__Bacteria;p__Cyanobacteria;c__Cyanobacteriia;o__Chloroplast;f__unidentified_Chloroplast;g__unidentified_Chloroplast	excluded	phylum_Cyanobacteria	17
tissue	ASV2403	k__Bacteria;p__Cyanobacteria;c__Cyanobacteriia;o__Chloroplast;f__unidentified_Chloroplast;g__unidentified_Chloroplast	excluded	phylum_Cyanobacteria	17
tissue	ASV2558	k__Bacteria;p__Cyanobacteria;c__Cyanobacteriia;o__Chloroplast;f__unidentified_Chloroplast;g__unidentified_Chloroplast	excluded	phylum_Cyanobacteria	15
tissue	ASV2586	k__Bacteria;p__Cyanobacteria;c__Cyanobacteriia;o__Chloroplast;f__unidentified_Chloroplast;g__unidentified_Chloroplast	excluded	phylum_Cyanobacteria	14
tissue	ASV2656	k__Bacteria;p__Cyanobacteria;c__Cyanobacteriia;o__Chloroplast;f__unidentified_Chloroplast;g__unidentified_Chloroplast	excluded	phylum_Cyanobacteria	13
tissue	ASV2658	k__Bacteria;p__Cyanobacteria;c__Cyanobacteriia;o__Chloroplast;f__unidentified_Chloroplast;g__unidentified_Chloroplast	excluded	phylum_Cyanobacteria	13
tissue	ASV2786	k__Bacteria;p__Cyanobacteria;c__Cyanobacteriia;o__Chloroplast;f__unidentified_Chloroplast;g__unidentified_Chloroplast	excluded	phylum_Cyanobacteria	12
tissue	ASV2798	k__Bacteria;p__Cyanobacteria;c__Cyanobacteriia;o__Chloroplast;f__unidentified_Chloroplast;g__unidentified_Chloroplast	excluded	phylum_Cyanobacteria	12
tissue	ASV2811	k__Bacteria;p__Cyanobacteria;c__Cyanobacteriia;o__Cyanobacteriales;f__Chroococcidiopsaceae;g__Aliterella;s__	excluded	phylum_Cyanobacteria	11
tissue	ASV2899	k__Bacteria;p__Cyanobacteria;c__Cyanobacteriia;o__Synechococcales;f__Cyanobiaceae;g__Cyanobium_PCC-6307	excluded	phylum_Cyanobacteria	10
tissue	ASV3202	k__Bacteria;p__Cyanobacteria;c__Cyanobacteriia;o__Chloroplast;f__unidentified_Chloroplast;g__unidentified_Chloroplast	excluded	phylum_Cyanobacteria	7
tissue	ASV3234	k__Bacteria;p__Cyanobacteria;c__Sericytochromatia;o__;f__;g__;s__	excluded	phylum_Cyanobacteria	0
tissue	ASV3282	k__Bacteria;p__Cyanobacteria;c__Cyanobacteriia;o__Cyanobacteriales;f__Nostocaceae	excluded	phylum_Cyanobacteria	6
tissue	ASV3339	k__Bacteria;p__Cyanobacteria;c__Vampirivibrionia;o__Obscuribacterales;f__Obscuribacteraceae;g__;s__	excluded	phylum_Cyanobacteria	0
tissue	ASV3376	k__Bacteria;p__Cyanobacteria;c__Cyanobacteriia;o__Chloroplast;f__unidentified_Chloroplast;g__unidentified_Chloroplast	excluded	phylum_Cyanobacteria	6
tissue	ASV3378	k__Bacteria;p__Cyanobacteria;c__Cyanobacteriia;o__Chloroplast;f__unidentified_Chloroplast;g__unidentified_Chloroplast	excluded	phylum_Cyanobacteria	6
tissue	ASV3726	k__Bacteria;p__Cyanobacteria;c__Cyanobacteriia;o__Chloroplast;f__unidentified_Chloroplast;g__unidentified_Chloroplast	excluded	phylum_Cyanobacteria	3
tissue	ASV3734	k__Bacteria;p__Cyanobacteria;c__Vampirivibrionia;o__Gastranaerophilales;f__;g__;s__	excluded	phylum_Cyanobacteria	3
tissue	ASV3895	k__Bacteria;p__Cyanobacteria;c__Cyanobacteriia;o__Chloroplast;f__unidentified_Chloroplast;g__unidentified_Chloroplast	excluded	phylum_Cyanobacteria	2
tissue	ASV3947	k__Bacteria;p__Cyanobacteria;c__Cyanobacteriia;o__Cyanobacteriales;f__Phormidiaceae;g__Phormidium_IAM_M-71;s__	excluded	phylum_Cyanobacteria	0
tissue	ASV3981	k__Bacteria;p__Cyanobacteria;c__Cyanobacteriia;o__Chloroplast;f__unidentified_Chloroplast;g__unidentified_Chloroplast	excluded	phylum_Cyanobacteria	2
wild	ASV0	k__Bacteria;p__Cyanobacteria;c__Cyanobacteriia;o__Chloroplast;f__unidentified_Chloroplast;g__unidentified_Chloroplast	excluded	phylum_Cyanobacteria	394,400
wild	ASV154	k__Bacteria;p__Cyanobacteria;c__Cyanobacteriia;o__Chloroplast;f__unidentified_Chloroplast;g__unidentified_Chloroplast	excluded	phylum_Cyanobacteria	492
wild	ASV165	k__Bacteria;p__Cyanobacteria;c__Cyanobacteriia;o__Chloroplast;f__unidentified_Chloroplast;g__unidentified_Chloroplast	excluded	phylum_Cyanobacteria	460
wild	ASV188	k__Bacteria;p__Cyanobacteria;c__Cyanobacteriia;o__Chloroplast;f__unidentified_Chloroplast;g__unidentified_Chloroplast	excluded	phylum_Cyanobacteria	410
wild	ASV243	k__Bacteria;p__Cyanobacteria;c__Cyanobacteriia;o__Chloroplast;f__unidentified_Chloroplast;g__unidentified_Chloroplast	excluded	phylum_Cyanobacteria	310
wild	ASV254	k__Bacteria;p__Cyanobacteria;c__Cyanobacteriia;o__Chloroplast;f__unidentified_Chloroplast;g__unidentified_Chloroplast	excluded	phylum_Cyanobacteria	292
wild	ASV289	k__Bacteria;p__Cyanobacteria;c__Cyanobacteriia;o__Chloroplast;f__unidentified_Chloroplast;g__unidentified_Chloroplast	excluded	phylum_Cyanobacteria	254
wild	ASV302	k__Bacteria;p__Cyanobacteria;c__Cyanobacteriia;o__Chloroplast;f__unidentified_Chloroplast;g__unidentified_Chloroplast	excluded	phylum_Cyanobacteria	241
wild	ASV322	k__Bacteria;p__Cyanobacteria;c__Cyanobacteriia;o__Chloroplast;f__unidentified_Chloroplast;g__unidentified_Chloroplast	excluded	phylum_Cyanobacteria	229
wild	ASV348	k__Bacteria;p__Cyanobacteria;c__Cyanobacteriia;o__Chloroplast;f__unidentified_Chloroplast;g__unidentified_Chloroplast	excluded	phylum_Cyanobacteria	206

wild	ASV379	k__Bacteria;p__Cyanobacteria;c__Cyanobacteriia;o__Chloroplast;f__unidentified_Chloroplast;g__unidentified_Chloroplast	excluded	phylum_Cyanobacteria	194
wild	ASV393	k__Bacteria;p__Cyanobacteria;c__Cyanobacteriia;o__Chloroplast;f__unidentified_Chloroplast;g__unidentified_Chloroplast	excluded	phylum_Cyanobacteria	187
wild	ASV398	k__Bacteria;p__Cyanobacteria;c__Cyanobacteriia;o__Chloroplast;f__unidentified_Chloroplast;g__unidentified_Chloroplast	excluded	phylum_Cyanobacteria	183
wild	ASV425	k__Bacteria;p__Cyanobacteria;c__Cyanobacteriia;o__Chloroplast;f__unidentified_Chloroplast;g__unidentified_Chloroplast	excluded	phylum_Cyanobacteria	171
wild	ASV438	k__Bacteria;p__Cyanobacteria;c__Cyanobacteriia;o__Chloroplast;f__unidentified_Chloroplast;g__unidentified_Chloroplast	excluded	phylum_Cyanobacteria	165
wild	ASV473	k__Bacteria;p__Cyanobacteria;c__Cyanobacteriia;o__Chloroplast;f__unidentified_Chloroplast;g__unidentified_Chloroplast	excluded	phylum_Cyanobacteria	151
wild	ASV477	k__Bacteria;p__Cyanobacteria;c__Cyanobacteriia;o__Chloroplast;f__unidentified_Chloroplast;g__unidentified_Chloroplast	excluded	phylum_Cyanobacteria	150
wild	ASV488	k__Bacteria;p__Cyanobacteria;c__Cyanobacteriia;o__Chloroplast;f__unidentified_Chloroplast;g__unidentified_Chloroplast	excluded	phylum_Cyanobacteria	146
wild	ASV539	k__Bacteria;p__Cyanobacteria;c__Cyanobacteriia;o__Chloroplast;f__unidentified_Chloroplast;g__unidentified_Chloroplast	excluded	phylum_Cyanobacteria	129
wild	ASV551	k__Bacteria;p__Cyanobacteria;c__Cyanobacteriia;o__Chloroplast;f__unidentified_Chloroplast;g__unidentified_Chloroplast	excluded	phylum_Cyanobacteria	128
wild	ASV558	k__Bacteria;p__Cyanobacteria;c__Cyanobacteriia;o__Chloroplast;f__unidentified_Chloroplast;g__unidentified_Chloroplast	excluded	phylum_Cyanobacteria	127
wild	ASV578	k__Bacteria;p__Cyanobacteria;c__Cyanobacteriia;o__Chloroplast;f__unidentified_Chloroplast;g__unidentified_Chloroplast	excluded	phylum_Cyanobacteria	123
wild	ASV627	k__Bacteria;p__Cyanobacteria;c__Cyanobacteriia;o__Chloroplast;f__unidentified_Chloroplast;g__unidentified_Chloroplast	excluded	phylum_Cyanobacteria	112
wild	ASV640	k__Bacteria;p__Cyanobacteria;c__Cyanobacteriia;o__Chloroplast;f__unidentified_Chloroplast;g__unidentified_Chloroplast	excluded	phylum_Cyanobacteria	109
wild	ASV644	k__Bacteria;p__Cyanobacteria;c__Cyanobacteriia;o__Chloroplast;f__unidentified_Chloroplast;g__unidentified_Chloroplast	excluded	phylum_Cyanobacteria	108
wild	ASV665	k__Bacteria;p__Cyanobacteria;c__Cyanobacteriia;o__Chloroplast;f__unidentified_Chloroplast;g__unidentified_Chloroplast	excluded	phylum_Cyanobacteria	105
wild	ASV666	k__Bacteria;p__Cyanobacteria;c__Cyanobacteriia;o__Chloroplast;f__unidentified_Chloroplast;g__unidentified_Chloroplast	excluded	phylum_Cyanobacteria	105
wild	ASV686	k__Bacteria;p__Cyanobacteria;c__Cyanobacteriia;o__Chloroplast;f__unidentified_Chloroplast;g__unidentified_Chloroplast	excluded	phylum_Cyanobacteria	100
wild	ASV691	k__Bacteria;p__Cyanobacteria;c__Cyanobacteriia;o__Chloroplast;f__unidentified_Chloroplast;g__unidentified_Chloroplast	excluded	phylum_Cyanobacteria	98
wild	ASV698	k__Bacteria;p__Cyanobacteria;c__Cyanobacteriia;o__Chloroplast;f__unidentified_Chloroplast;g__unidentified_Chloroplast	excluded	phylum_Cyanobacteria	97
wild	ASV726	k__Bacteria;p__Cyanobacteria;c__Cyanobacteriia;o__Chloroplast;f__unidentified_Chloroplast;g__unidentified_Chloroplast	excluded	phylum_Cyanobacteria	93
wild	ASV796	k__Bacteria;p__Cyanobacteria;c__Cyanobacteriia;o__Chloroplast;f__unidentified_Chloroplast;g__unidentified_Chloroplast	excluded	phylum_Cyanobacteria	84
wild	ASV806	k__Bacteria;p__Cyanobacteria;c__Cyanobacteriia;o__Chloroplast;f__unidentified_Chloroplast;g__unidentified_Chloroplast	excluded	phylum_Cyanobacteria	83
wild	ASV838	k__Bacteria;p__Cyanobacteria;c__Cyanobacteriia;o__Chloroplast;f__unidentified_Chloroplast;g__unidentified_Chloroplast	excluded	phylum_Cyanobacteria	78
wild	ASV874	k__Bacteria;p__Cyanobacteria;c__Cyanobacteriia;o__Chloroplast;f__unidentified_Chloroplast;g__unidentified_Chloroplast	excluded	phylum_Cyanobacteria	75
wild	ASV961	k__Bacteria;p__Cyanobacteria;c__Cyanobacteriia;o__Chloroplast;f__unidentified_Chloroplast;g__unidentified_Chloroplast	excluded	phylum_Cyanobacteria	65
wild	ASV1052	k__Bacteria;p__Cyanobacteria;c__Cyanobacteriia;o__Chloroplast;f__unidentified_Chloroplast;g__unidentified_Chloroplast	excluded	phylum_Cyanobacteria	58
wild	ASV1066	k__Bacteria;p__Cyanobacteria;c__Cyanobacteriia;o__Chloroplast;f__unidentified_Chloroplast;g__unidentified_Chloroplast	excluded	phylum_Cyanobacteria	57
wild	ASV1209	k__Bacteria;p__Cyanobacteria;c__Cyanobacteriia;o__Chloroplast;f__unidentified_Chloroplast;g__unidentified_Chloroplast	excluded	phylum_Cyanobacteria	50
wild	ASV1214	k__Bacteria;p__Cyanobacteria;c__Cyanobacteriia;o__Chloroplast;f__unidentified_Chloroplast;g__unidentified_Chloroplast	excluded	phylum_Cyanobacteria	49
wild	ASV1230	k__Bacteria;p__Cyanobacteria;c__Cyanobacteriia;o__Chloroplast;f__unidentified_Chloroplast;g__unidentified_Chloroplast	excluded	phylum_Cyanobacteria	49
wild	ASV1316	k__Bacteria;p__Cyanobacteria;c__Cyanobacteriia;o__Chloroplast;f__unidentified_Chloroplast;g__unidentified_Chloroplast	excluded	phylum_Cyanobacteria	45
wild	ASV1355	k__Bacteria;p__Cyanobacteria;c__Cyanobacteriia;o__Chloroplast;f__unidentified_Chloroplast;g__unidentified_Chloroplast	excluded	phylum_Cyanobacteria	44
wild	ASV1359	k__Bacteria;p__Cyanobacteria;c__Cyanobacteriia;o__Chloroplast;f__unidentified_Chloroplast;g__unidentified_Chloroplast	excluded	phylum_Cyanobacteria	43
wild	ASV1371	k__Bacteria;p__Cyanobacteria;c__Cyanobacteriia;o__Chloroplast;f__unidentified_Chloroplast;g__unidentified_Chloroplast	excluded	phylum_Cyanobacteria	43
wild	ASV1385	k__Bacteria;p__Cyanobacteria;c__Cyanobacteriia;o__Chloroplast;f__unidentified_Chloroplast;g__unidentified_Chloroplast	excluded	phylum_Cyanobacteria	42
wild	ASV1496	k__Bacteria;p__Cyanobacteria;c__Cyanobacteriia;o__Chloroplast;f__unidentified_Chloroplast;g__unidentified_Chloroplast	excluded	phylum_Cyanobacteria	38
wild	ASV1588	k__Bacteria;p__Cyanobacteria;c__Cyanobacteriia;o__Chloroplast;f__unidentified_Chloroplast;g__unidentified_Chloroplast	excluded	phylum_Cyanobacteria	35
wild	ASV1589	k__Bacteria;p__Cyanobacteria;c__Cyanobacteriia;o__Chloroplast;f__unidentified_Chloroplast;g__unidentified_Chloroplast	excluded	phylum_Cyanobacteria	35
wild	ASV1624	k__Bacteria;p__Cyanobacteria;c__Cyanobacteriia;o__Chloroplast;f__unidentified_Chloroplast;g__unidentified_Chloroplast	excluded	phylum_Cyanobacteria	34

wild	ASV1625	k__Bacteria;p__Cyanobacteria;c__Cyanobacteriia;o__Chloroplast;f__unidentified_Chloroplast;g__unidentified_Chloroplast	excluded	phylum_Cyanobacteria	34
wild	ASV1802	k__Bacteria;p__Cyanobacteria;c__Cyanobacteriia;o__Chloroplast;f__unidentified_Chloroplast;g__unidentified_Chloroplast	excluded	phylum_Cyanobacteria	30
wild	ASV1901	k__Bacteria;p__Cyanobacteria;c__Cyanobacteriia;o__Chloroplast;f__unidentified_Chloroplast;g__unidentified_Chloroplast	excluded	phylum_Cyanobacteria	28
wild	ASV1966	k__Bacteria;p__Cyanobacteria;c__Cyanobacteriia;o__Leptolyngbyales;f__Leptolyngbyaceae;g__MTP1;s__	excluded	phylum_Cyanobacteria	27
wild	ASV2138	k__Bacteria;p__Cyanobacteria;c__Cyanobacteriia;o__Chloroplast;f__unidentified_Chloroplast;g__unidentified_Chloroplast	excluded	phylum_Cyanobacteria	24
wild	ASV2200	k__Bacteria;p__Cyanobacteria;c__Cyanobacteriia;o__Chloroplast;f__unidentified_Chloroplast;g__unidentified_Chloroplast	excluded	phylum_Cyanobacteria	23
wild	ASV2205	k__Bacteria;p__Cyanobacteria;c__Cyanobacteriia;o__Chloroplast;f__unidentified_Chloroplast;g__unidentified_Chloroplast	excluded	phylum_Cyanobacteria	23
wild	ASV2334	k__Bacteria;p__Cyanobacteria;c__Cyanobacteriia;o__Chloroplast;f__unidentified_Chloroplast;g__unidentified_Chloroplast	excluded	phylum_Cyanobacteria	21
wild	ASV2360	k__Bacteria;p__Cyanobacteria;c__Cyanobacteriia;o__Chloroplast;f__unidentified_Chloroplast;g__unidentified_Chloroplast	excluded	phylum_Cyanobacteria	20
wild	ASV2396	k__Bacteria;p__Cyanobacteria;c__Cyanobacteriia;o__Chloroplast;f__unidentified_Chloroplast;g__unidentified_Chloroplast	excluded	phylum_Cyanobacteria	20
wild	ASV2598	k__Bacteria;p__Cyanobacteria;c__Cyanobacteriia;o__Chloroplast;f__unidentified_Chloroplast;g__unidentified_Chloroplast	excluded	phylum_Cyanobacteria	18
wild	ASV2654	k__Bacteria;p__Cyanobacteria;c__Cyanobacteriia;o__Chloroplast;f__unidentified_Chloroplast;g__unidentified_Chloroplast	excluded	phylum_Cyanobacteria	17
wild	ASV2655	k__Bacteria;p__Cyanobacteria;c__Cyanobacteriia;o__Chloroplast;f__unidentified_Chloroplast;g__unidentified_Chloroplast	excluded	phylum_Cyanobacteria	17
wild	ASV2677	k__Bacteria;p__Cyanobacteria;c__Cyanobacteriia;o__Chloroplast;f__unidentified_Chloroplast;g__unidentified_Chloroplast	excluded	phylum_Cyanobacteria	17
wild	ASV2838	k__Bacteria;p__Cyanobacteria;c__Cyanobacteriia;o__Chloroplast;f__unidentified_Chloroplast;g__unidentified_Chloroplast	excluded	phylum_Cyanobacteria	15
wild	ASV2858	k__Bacteria;p__Cyanobacteria;c__Cyanobacteriia;o__Chloroplast;f__unidentified_Chloroplast;g__unidentified_Chloroplast	excluded	phylum_Cyanobacteria	15
wild	ASV3043	k__Bacteria;p__Cyanobacteria;c__Cyanobacteriia;o__Chloroplast;f__unidentified_Chloroplast;g__unidentified_Chloroplast	excluded	phylum_Cyanobacteria	13
wild	ASV3482	k__Bacteria;p__Cyanobacteria;c__Cyanobacteriia;o__Chloroplast;f__unidentified_Chloroplast;g__unidentified_Chloroplast	excluded	phylum_Cyanobacteria	11
wild	ASV3578	k__Bacteria;p__Cyanobacteria;c__Cyanobacteriia;o__Chloroplast;f__unidentified_Chloroplast;g__unidentified_Chloroplast	excluded	phylum_Cyanobacteria	10
wild	ASV3885	k__Bacteria;p__Cyanobacteria;c__Cyanobacteriia;o__Chloroplast;f__unidentified_Chloroplast;g__unidentified_Chloroplast	excluded	phylum_Cyanobacteria	9
wild	ASV3895	k__Bacteria;p__Cyanobacteria;c__Sericytochromatia;o__;f__;g__;s__	excluded	phylum_Cyanobacteria	9
wild	ASV3915	k__Bacteria;p__Cyanobacteria;c__Cyanobacteriia;o__Chloroplast;f__unidentified_Chloroplast;g__unidentified_Chloroplast	excluded	phylum_Cyanobacteria	9
wild	ASV3980	k__Bacteria;p__Cyanobacteria;c__Cyanobacteriia;o__Chloroplast;f__unidentified_Chloroplast;g__unidentified_Chloroplast	excluded	phylum_Cyanobacteria	8
wild	ASV4230	k__Bacteria;p__Cyanobacteria;c__Cyanobacteriia;o__Chloroplast;f__unidentified_Chloroplast;g__unidentified_Chloroplast	excluded	phylum_Cyanobacteria	8
wild	ASV4367	k__Bacteria;p__Cyanobacteria;c__Cyanobacteriia;o__Chloroplast;f__unidentified_Chloroplast;g__unidentified_Chloroplast	excluded	phylum_Cyanobacteria	7
wild	ASV4379	k__Bacteria;p__Cyanobacteria;c__Cyanobacteriia;o__Cyanobacteriales;f__Chroococcidiopsaceae;g__Crinalium_SAG_22.89;s__	excluded	phylum_Cyanobacteria	7
wild	ASV4553	k__Bacteria;p__Cyanobacteria;c__Cyanobacteriia;o__Chloroplast;f__unidentified_Chloroplast;g__unidentified_Chloroplast	excluded	phylum_Cyanobacteria	7
wild	ASV5287	k__Bacteria;p__Cyanobacteria;c__Vampirivibrionia;o__Gastranaerophilales;f__;g__;s__	excluded	phylum_Cyanobacteria	5
wild	ASV5300	k__Bacteria;p__Cyanobacteria;c__Cyanobacteriia;o__Chloroplast;f__unidentified_Chloroplast;g__unidentified_Chloroplast	excluded	phylum_Cyanobacteria	5
wild	ASV5345	k__Bacteria;p__Cyanobacteria;c__Cyanobacteriia;o__Chloroplast;f__unidentified_Chloroplast;g__unidentified_Chloroplast	excluded	phylum_Cyanobacteria	5
wild	ASV5603	k__Bacteria;p__Cyanobacteria;c__Cyanobacteriia;o__Synechococcales;f__Cyanobiaceae;g__Synechococcus_CC9902	excluded	phylum_Cyanobacteria	4
wild	ASV6262	k__Bacteria;p__Cyanobacteria;c__Vampirivibrionia;o__Gastranaerophilales;f__;g__;s__	excluded	phylum_Cyanobacteria	3
wild	ASV6902	k__Bacteria;p__Cyanobacteria;c__Cyanobacteriia;o__Chloroplast;f__unidentified_Chloroplast;g__unidentified_Chloroplast	excluded	phylum_Cyanobacteria	3
wild	ASV6941	k__Bacteria;p__Cyanobacteria;c__Cyanobacteriia;o__Chloroplast;f__unidentified_Chloroplast;g__unidentified_Chloroplast	excluded	phylum_Cyanobacteria	3
wild	ASV6989	k__Bacteria;p__Cyanobacteria;c__Vampirivibrionia;o__Gastranaerophilales;f__;g__;s__	excluded	phylum_Cyanobacteria	2
wild	ASV7058	k__Bacteria;p__Cyanobacteria;c__Cyanobacteriia;o__Chloroplast;f__unidentified_Chloroplast;g__unidentified_Chloroplast	excluded	phylum_Cyanobacteria	2
wild	ASV7336	k__Bacteria;p__Cyanobacteria;c__Cyanobacteriia;o__Chloroplast;f__unidentified_Chloroplast;g__unidentified_Chloroplast	excluded	phylum_Cyanobacteria	2
wild	ASV7530	k__Bacteria;p__Cyanobacteria;c__Cyanobacteriia;o__Chloroplast;f__unidentified_Chloroplast;g__unidentified_Chloroplast	excluded	phylum_Cyanobacteria	2
wild	ASV7535	k__Bacteria;p__Cyanobacteria;c__Cyanobacteriia;o__Chloroplast;f__unidentified_Chloroplast;g__unidentified_Chloroplast	excluded	phylum_Cyanobacteria	2
wild	ASV7630	k__Bacteria;p__Cyanobacteria;c__Cyanobacteriia;o__Chloroplast;f__unidentified_Chloroplast;g__unidentified_Chloroplast	excluded	phylum_Cyanobacteria	2

wild	ASV7632	k__Bacteria;p__Cyanobacteria;c__Cyanobacteriia;o__Chloroplast;f__unidentified_Chloroplast;g__unidentified_Chloroplast	excluded	phylum_Cyanobacteria	2
wild	ASV7634	k__Bacteria;p__Cyanobacteria;c__Cyanobacteriia;o__Chloroplast;f__unidentified_Chloroplast;g__unidentified_Chloroplast	excluded	phylum_Cyanobacteria	2
wild	ASV7672	k__Bacteria;p__Cyanobacteria;c__Cyanobacteriia;o__Chloroplast;f__unidentified_Chloroplast;g__unidentified_Chloroplast	excluded	phylum_Cyanobacteria	2
wild	ASV7753	k__Bacteria;p__Cyanobacteria;c__Cyanobacteriia;o__Chloroplast;f__unidentified_Chloroplast;g__unidentified_Chloroplast	excluded	phylum_Cyanobacteria	2

Notes

Filtering rule All listed ASVs were assigned to phylum Cyanobacteria and were excluded. unidentified_Chloroplast annotations occur within that phylum. SILVA v138.1 was used for taxonomic assignment in all three projects, with a classify-sklearn confidence threshold of 0.7.

Excluded ASVs The table lists 85 laboratory, 56 tissue/frass and 94 field ASVs. Dataset-specific ASV identifiers are not unique across sequencing projects.

Sequence counts Sequences removed are summed across manuscript samples before rarefaction. Dataset totals reconcile with the per-sample removed counts in Table S1.

Filtering interpretation This taxonomic rule reduces plant-associated chloroplast sequences. It is not a negative-control-based contaminant detection analysis and does not establish the origins of other detected bacterial genera.

Source records Company ASV taxonomy tables and the recorded Cyanobacteria filtering lists.

Table S4. Overall Bray–Curtis PERMANOVA and PERMDISP results

dataset	n_samples	n_groups	df_between	df_residual	pseudo_F	R2	p_value	permutations	PERMDISP_F	PERMDISP_p	rarefaction_depth	removed_Cyanobacteria_ASVs	rarefaction_seed	PERMANOVA_seed	PERMDISP_seed	PERMDISP_Lingoes_constant
lab	40	8	7	32	3.820742	0.455275	0.001	999	7.161901	0.001	38,220	85	20261106	20262106	20262107	0.040114
tissue	16	4	3	12	2.475305	0.382268	0.008	999	6.844708	0.022	46,164	56	20261308	20262308	20262309	0.000000
wild	25	5	4	20	0.835576	0.143186	0.669	999	0.226866	0.920	3,745	94	20261207	20262207	20262208	0.015143

Notes	
**Analysis unit**	Each sample is one pooled biological replicate and one unit of analysis. Laboratory developmental-stage/adult-sex groups and field instars each have five pooled biological replicates. Each tissue/frass type has four pooled biological replicates. Individuals within a pool are not counted as separate replicates.
**Dataset groups**	lab: eight developmental-stage/adult-sex groups, with five pooled replicates per group. tissue: four tissue/frass types, with four pooled replicates per type. wild: five instars, with five pooled replicates per instar.
**Female lineages**	Five independent female lineages were established for the laboratory developmental-stage/adult-sex comparison. Each group contains one pooled sample from each lineage. Individuals within a pool originate from the same female lineage, and the five pools within a group originate from five different female lineages.
**PERMANOVA**	Bray–Curtis analyses use the original integer ASV counts after exclusion of Cyanobacteria-assigned ASVs. Counts were rarefied once without replacement to 38,220 (lab), 46,164 (tissue), or 3,745 (wild) sequences per pooled sample. PERMANOVA used one factor, 999 unrestricted permutations, and scikit-bio v0.7.4.
**PERMDISP**	PERMDISP used spatial medians and 999 permutations. Lingoes correction was applied only for dispersion calculations where negative eigenvalues occurred. PERMANOVA used uncorrected Bray–Curtis dissimilarities. Random seeds and Lingoes constants are recorded in Table S4.
**Ordination**	The manuscript PCoA figures use weighted UniFrac distances. Tables S4 and S5 report the separate inferential analyses based on Bray–Curtis dissimilarities.
**Pairwise results**	Pairwise PERMANOVA results and within-dataset Benjamini–Hochberg FDR correction are provided in Table S5.
**Alpha diversity**	Shannon and Chao1 comparisons used the Kruskal–Wallis test followed by Dunn's post hoc test with FDR correction. Letter groups follow the adjusted pairwise comparisons. Tables S4 and S5 do not contain alpha-diversity tests.
**Precision**	Full numeric values are retained in cells. Display formatting rounds floating-point values only. With 999 permutations, the minimum p-value resolution is 0.001.
**Source records**	Existing verified Cyanobacteria-filtered Bray–Curtis analysis records generated on 5 October 2026 using scikit-bio v0.7.4.

Table S5. Pairwise Bray–Curtis PERMANOVA with FDR correction

dataset	group1	group2	n_samples	pseudo_F	R2	p_raw	p_BH_FDR	permutations	seed	significant_FDR_0.05
lab	L1	L2	10	4.578059	0.363972	0.011	0.023692	999	20262116	TRUE
lab	L1	L3	10	17.992118	0.692214	0.009	0.023333	999	20262117	TRUE
lab	L1	L4	10	1.900836	0.191987	0.048	0.064000	999	20262118	FALSE
lab	L1	L5	10	3.824582	0.323443	0.009	0.023333	999	20262119	TRUE
lab	L1	Pu	10	1.939561	0.195135	0.074	0.094182	999	20262120	FALSE
lab	L1	Ma	10	2.063069	0.205014	0.015	0.024706	999	20262121	TRUE
lab	L1	Fe	10	2.487494	0.237187	0.018	0.028000	999	20262122	TRUE
lab	L2	L3	10	16.670640	0.675728	0.013	0.024500	999	20262123	TRUE
lab	L2	L4	10	1.895890	0.191584	0.087	0.105913	999	20262124	FALSE
lab	L2	L5	10	1.527280	0.160306	0.150	0.175000	999	20262125	FALSE
lab	L2	Pu	10	2.998733	0.272643	0.008	0.023333	999	20262126	TRUE
lab	L2	Ma	10	5.556799	0.409890	0.010	0.023333	999	20262127	TRUE
lab	L2	Fe	10	5.882656	0.423741	0.010	0.023333	999	20262128	TRUE
lab	L3	L4	10	4.681777	0.369174	0.046	0.064000	999	20262129	FALSE
lab	L3	L5	10	4.646995	0.367439	0.006	0.023333	999	20262130	TRUE
lab	L3	Pu	10	4.096058	0.338627	0.034	0.050105	999	20262131	FALSE
lab	L3	Ma	10	24.232539	0.751804	0.008	0.023333	999	20262132	TRUE
lab	L3	Fe	10	25.605101	0.761941	0.014	0.024500	999	20262133	TRUE
lab	L4	L5	10	0.658833	0.076088	0.782	0.782000	999	20262134	FALSE
lab	L4	Pu	10	0.830955	0.094096	0.578	0.622462	999	20262135	FALSE
lab	L4	Ma	10	2.500348	0.238120	0.008	0.023333	999	20262136	TRUE
lab	L4	Fe	10	2.601184	0.245367	0.014	0.024500	999	20262137	TRUE
lab	L5	Pu	10	1.254928	0.135596	0.285	0.319200	999	20262138	FALSE
lab	L5	Ma	10	4.677162	0.368944	0.008	0.023333	999	20262139	TRUE
lab	L5	Fe	10	4.876987	0.378737	0.006	0.023333	999	20262140	TRUE
lab	Pu	Ma	10	2.598915	0.245206	0.008	0.023333	999	20262141	TRUE
lab	Pu	Fe	10	2.836100	0.261727	0.010	0.023333	999	20262142	TRUE
lab	Ma	Fe	10	0.804046	0.091327	0.694	0.719704	999	20262143	FALSE
tissue	ML	EL	8	3.963395	0.397796	0.027	0.066000	999	20262318	FALSE
tissue	ML	FL	8	3.920187	0.395173	0.033	0.066000	999	20262319	FALSE
tissue	ML	OL	8	1.283525	0.176223	0.233	0.246000	999	20262320	FALSE
tissue	EL	FL	8	3.154491	0.344584	0.061	0.091500	999	20262321	FALSE
tissue	EL	OL	8	1.218204	0.168768	0.246	0.246000	999	20262322	FALSE
tissue	FL	OL	8	2.008992	0.250842	0.030	0.066000	999	20262323	FALSE
wild	WL1	WL2	10	0.592138	0.068916	0.697	0.774444	999	20262217	FALSE
wild	WL1	WL3	10	1.199925	0.130428	0.211	0.761250	999	20262218	FALSE
wild	WL1	WL4	10	1.045918	0.115623	0.332	0.761250	999	20262219	FALSE

wild	WL1	WL5	10	0.904393	0.101567	0.325	0.761250	999	20262220	FALSE
wild	WL2	WL3	10	0.904950	0.101623	0.501	0.761250	999	20262221	FALSE
wild	WL2	WL4	10	1.006845	0.111787	0.356	0.761250	999	20262222	FALSE
wild	WL2	WL5	10	0.421791	0.050083	0.609	0.761250	999	20262223	FALSE
wild	WL3	WL4	10	0.472775	0.055799	0.987	0.987000	999	20262224	FALSE
wild	WL3	WL5	10	0.833823	0.094390	0.564	0.761250	999	20262225	FALSE
wild	WL4	WL5	10	0.906050	0.101734	0.414	0.761250	999	20262226	FALSE

Notes

**Analysis unit**	n_samples is the total number of pooled biological replicates in the two groups being compared: 10 for laboratory or field pairs, and 8 for tissue/frass pairs. It is not the number of insects contributing to the pools.
**Pool size**	For laboratory developmental-stage/adult-sex and field-instar samples, each replicate contains midguts from 5–15 individuals. Pool size varies with body size to obtain sufficient material and DNA. For the fifth-instar tissue comparison, each midgut, epidermis or other-internal-tissue replicate pools the corresponding material from five larvae. Each frass replicate pools frass from ten larvae. Different sample types originate from different insects.
**Female lineages**	Five independent female lineages were established for the laboratory developmental-stage/adult-sex comparison. Each group contains one pooled sample from each lineage. Individuals within a pool originate from the same female lineage, and the five pools within a group originate from five different female lineages.
**Groups**	L1–L5: first- to fifth-instar laboratory larvae. Pu: pupae. Ma: male adults. Fe: female adults. WL1–WL5: first- to fifth-instar field larvae. ML/ML5: midgut. EL/EL5: epidermis. OL/OL5: other internal tissues. FL/FL5: frass. Company WL2–WL6 correspond to manuscript WL1–WL5.
**PERMANOVA**	Bray–Curtis analyses use the original integer ASV counts after exclusion of Cyanobacteria-assigned ASVs. Counts were rarefied once without replacement to 38,220 (lab), 46,164 (tissue), or 3,745 (wild) sequences per pooled sample. PERMANOVA used one factor, 999 unrestricted permutations, and scikit-bio v0.7.4.
**Multiple comparisons**	Benjamini–Hochberg FDR correction was applied separately within each dataset across 28 laboratory, 6 tissue/frass and 10 field comparisons. significant_FDR_0.05 is TRUE only when p_BH_FDR < 0.05.
**Overall and dispersion**	Overall PERMANOVA and PERMDISP results, rarefaction seeds, dispersion seeds and Lingoes constants are provided in Table S4.
**Precision**	Full numeric values are retained in cells. FDR-adjusted p-values are displayed to six decimal places to distinguish values near 0.05. With 999 permutations, the minimum raw p-value resolution is 0.001.
**Source records**	Existing verified Cyanobacteria-filtered pairwise Bray–Curtis analysis records generated on 5 October 2026 using scikit-bio v0.7.4.
